# Streptoglycerides E–H, Unsaturated Polyketides from the Marine-Derived Bacterium *Streptomyces* *specialis* and Their Anti-Inflammatory Activity

**DOI:** 10.3390/md20010044

**Published:** 2022-01-01

**Authors:** Hee Jae Shin, Chang-Su Heo, Cao Van Anh, Yeo Dae Yoon, Jong Soon Kang

**Affiliations:** 1 Marine Natural Products Chemistry Laboratory, Korea Institute of Ocean Science and Technology, 385 Haeyang-ro, Yeongdo-gu, Busan 49111, Korea; science30@kiost.ac.kr (C.-S.H.); caovananh@kiost.ac.kr (C.V.A.); 2 Department of Marine Biotechnology, University of Science and Technology (UST), 217 Gajungro, Yuseong-gu, Daejeon 34113, Korea; 3 Laboratory Animal Resource Center, Korea Research Institute of Bioscience and Biotechnology, 30 Yeongudanjiro, Cheongju 28116, Korea; yunyd76@kribb.re.kr (Y.D.Y.); kanjon@kribb.re.kr (J.S.K.)

**Keywords:** marine-derived bacterium, *Streptomyces specialis*, streptoglycerides, anti-inflammatory

## Abstract

Four new streptoglycerides E–H (**1**–**4**), with a rare 6/5/5/-membered ring system, were isolated from a marine-derived actinomycete *Streptomyces specialis*. The structures of **1**–**4** were elucidated by detailed analysis of HRESIMS, 1D and 2D NMR data and ECD spectra as well as comparison of their spectroscopic data with those reported in literature. Compounds **1**–**4** showed significant anti-inflammatory activity by inhibiting lipopolysaccharide (LPS)-induced nitric oxide (NO) production in Raw 264.7 cells with IC_50_ values ranging from 3.5 to 10.9 µM. Especially, **2** suppressed mRNA expression levels of iNOS and IL-6 without cytotoxicity.

## 1. Introduction

Inflammation is a normal defense mechanism that occurs to cope with tissue damage and microbial infection [1]. When tissues are exposed to LPS, NO and cytokines such as interleukins (IL-6 and IL-1β), and tumor necrosis factor-α (TNF-α) are produced naturally [2,3]. However, the uncontrolled cytokine overproduction promotes a variety of diseases [4]. NO and interleukins play a central role in the pathogenesis of inflammation owing to overproduction under abnormal physiological conditions [5,6]. Therefore, NO and interleukins are considered as a critical indicator for developing drugs for inflammatory diseases [7,8].

Up to now, there have been many reports on marine natural products with anti-inflammatory activity [9,10,11]. Marine organisms inhabiting extreme environments have adopted unique survival strategies for growing and reproducing under hostile conditions, biosynthesizing molecules valuable for pharmaceutical applications [12,13]. Marine *Streptomyces* are an especially attractive source for exploring marine natural products as they produce various kinds of secondary metabolites with potent biological activities [14,15].

**Figure 1:**
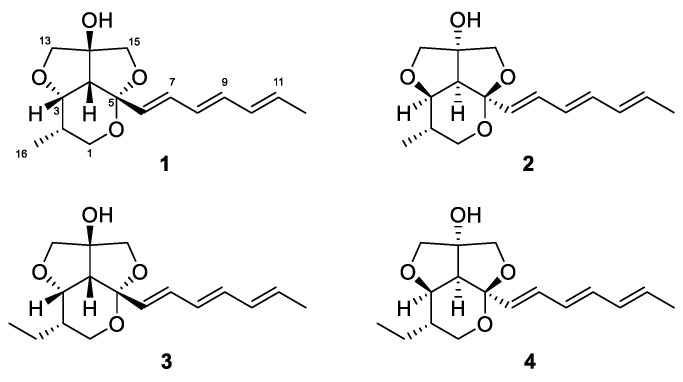
Structures of streptoglycerides E–H (**1**–**4**).

Recently, we reported streptoglycerides A–D with a quite rare 6/5/5 heterocyclic scaffold and anti-inflammatory activity isolated from a strain of marine-derived *Streptomyces* sp. [16]. During our ongoing investigation of secondary metabolites from marine microorganisms, we encountered another strain, designated as *Streptomyces specialis* 208DD-067, isolated from a sediment sample collected off Dokdo, South Korea, which produced new derivatives of streptoglycerides (**1**–**4**). Herein, we report the isolation, structure elucidation and anti-inflammatory activity of streptoglycerides E–H (**1**–**4**) (Figure 1).

## 2. Results and Discussion

### 2.1. Structural Elucidation

Compound **1** was isolated as a white powder. The molecular formula of **1** was determined to be C_16_H_22_O_4_ by combined analysis of HRESIMS (*m/z* 301.1417 [M+Na]^+^, calcd. for C_16_H_22_O_4_Na 301.1416) and NMR data. The ^1^H NMR spectra of **1** displayed signals of two methyl groups at *δ*_H_ 0.94 (d, *J* = 7.0, H_3_-16) and 1.76 (d, *J* = 7.0, H_3_-12); a methine at *δ*_H_ 2.41 (d, *J* = 7.0, H-4); six oxygenated methylenes at *δ*_H_ 3.36-4.00; an oxygenated methine at *δ*_H_ 4.11 (dd, *J* = 7.0, 3.1, H-3) and six olefinic protons at *δ*_H_ 5.66–6.40 (Table 1). The ^13^C NMR and HSQC spectra of **1** exhibited the presence of two methyl carbons at *δ*_C_ 13.0 and 18.4; three oxygenated methylene carbons at *δ*_C_ 62.3, 78.4 and 79.7; an oxygenated methine carbon at *δ*_C_ 80.0; eight methine carbons at *δ*_C_ 32.1, 54.3, 130.3, 131.5, 131.8, 132.9, 136.0 and 133.6; and two nonprotonated carbons at *δ*_C_ 87.4 and 106.1 (Table 1).

**Figure 2:**
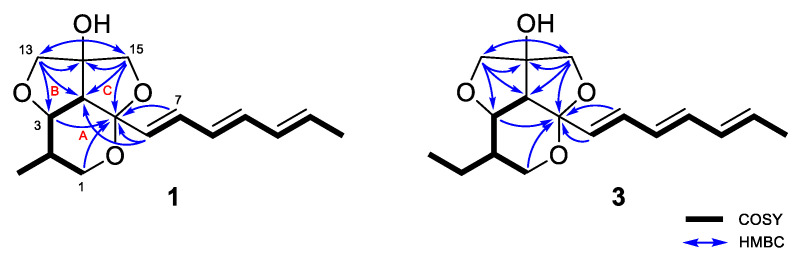
Key ^1^H-^1^H COSY and HMBC correlations for streptoglycerides E (**1**) and G (**3**).

The planar structure of **1** was elucidated by detailed analysis of ^1^H-^1^H COSY and HMBC data. The structure of ring A was determined as a 2,3,4-substituted-5-methyl-tetrahydropyran by the continuous COSY correlations from H-1a,b to H-4 through H-2, H-3, and H_3_-16 and the HMBC correlations from H-1a,b to C-5 (Figure 2). The HMBC correlations from H-13a,b to C-14 and C-15; and H-15a,b to C-13 and C-14 and their chemical shifts of *δ*_C_ 79.7 (C-13), 87.4 (C-14), and 78.4 (C-15) identified a partial structure corresponding to a 2-C-substituted glycerol moiety. The HMBC correlations from H-13a,b to C-3 and C-4 and from H-15a,b to C-4 and C-5 confirmed the connection of the glycerol moiety to the ring A via ether linkages of C-3/C-13 and C-5/C15 and the linkage between C-4 and C-14 was also determined by the HMBC correlations from H-13a,b to C-4 and C-14 and H-15a,b to C-4 and C-14. The presence of a triene side chain was determined by the continuous COSY correlations from H-6 to H_3_-12 and the connection of the side chain to the ring A at C-5 was confirmed by the HMBC correlations from H-6 to C-4 and C-5 and from H-7 to C-5. Thus, the planar structure of **1** was determined as 2-methylstreptoglyceride B which is closely related to streptoglyceride B, recently isolated from *Streptomyces* sp. [16].

**Figure 3:**
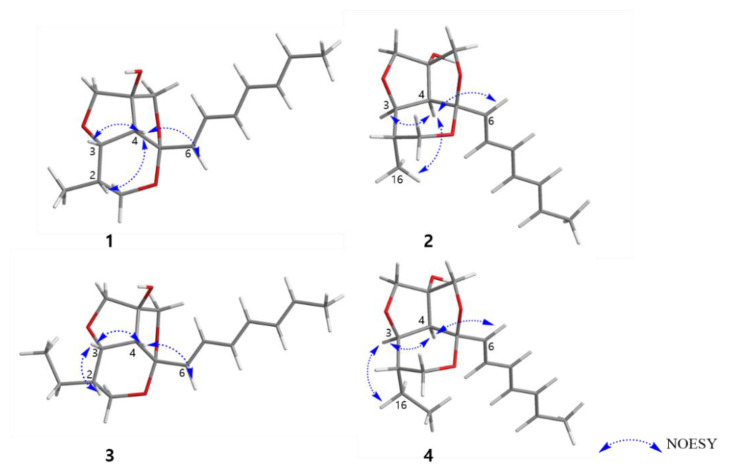
Key NOESY correlations of **1**–**4**.

The relative configuration of **1** was determined by NOESY correlations and coupling constant analysis (Figure 3). The geometries of double bonds in the triene chain were deduced as *E*-configuration on the basis of their large coupling constants (*J* ≥14 Hz, Table 1). The NOESY correlations from H-4 to H-2, H-3, H-6, and H-7 suggested that these protons had a cofacial relationship. The coupling constant (7.0 Hz) for H-3 and H-4 also indicated these two protons are located as a *syn*-arrangement.

**Figure 4:**
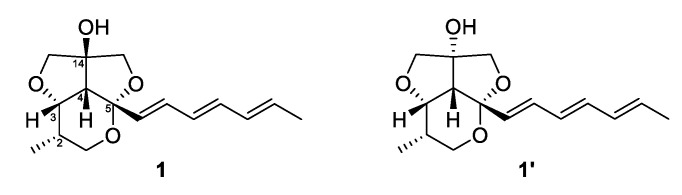
Structures of **1** and **1’**.

Furthermore, the fact that the furofuran ring system (rings B and C) is a rigid structure and always has a *cis*-fused relationship, on the basis of general stereochemical considerations, and the lack of NOE correlations from H-6 to H-13b and H-15b allowed us to determine the relative configuration of C-14 (OH-14 and H-4 had a *cis*-relationship) [17,18]. Therefore, the relative configuration of C-2, C-3, C-4, C-5 was determined as 2S*, 3S*, 4R*, 5S* as depicted in Figure 3. To further confirm this fact, the 3D models and conformational analysis of two possible relative configurations of **1** (2S*, 3S*, 4R*, 5S*, 14S*) and **1**’ (2S*, 3S*, 4R*, 5S*, 14R*) were built by Conflex program and their ^1^H and ^13^C chemical shifts were calculated by Gaussian software. Using both ^1^ H and ^13^C NMR chemical shifts in the DP4+ probability output resulted in a 100% preference for the **1** diastereomer over the **1**’ diastereomer (Figure 4, Figure S33 in the supplementary). This result also supported the fact that H-4 and OH-14 are cofacial. Thus, there were only two possible absolute configurations of the 6/5/5 tricyclic ring system for **1** as **1A** (2*S*, 3*S*, 4*R*, 5*S*, 14*S*) or **1B** (2*R*, *3R*, 4*S*, 5*R*, 14*R*).

**Figure 5:**
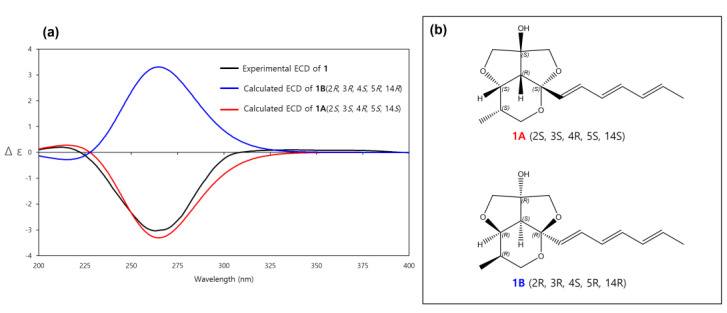
(**a**) Experimental and calculated ECD spectra of **1A** and **1B**. (**b**) Absolute configurations of **1A** (2*S*, 3*S*, 4*R*, 5*S*, 14*S*) and its enantiomer **1B** (2*R*, 3*R*, 4*S*, 5*R*, 14*R*).

The absolute configuration of **1** was determined by ECD spectrum calculations. The theoretical ECD spectra of **1A** (2*S*, 3*S*, 4*R*, 5*S*, 14*S*) and its enantiomer **1B** (2*R*, 3*R*, 4*S*, 5*R*, 14*R*) were generated by the Gaussian 16 program. The experimental ECD spectrum of **1** showed a good agreement with the calculated ECD spectrum of **1A** (Figure 5). Therefore, the absolute configuration of **1** was determined as 2*S*, 3*S*, 4*R*, 5*S*, 14*S,* which was same to other natural products with the 6/5/5 tricyclic ring skeleton (diocollettine A, streptoglycerides A–D, and bafilomycins P-Q) [16,17,19]. Thus, **1** was determined as a new derivative of streptoglyceride B and named streptoglyceride E. 

Compound **2** was also isolated as a white powder. The molecular formula of **2** was determined to be the same as that of **1**, C_16_H_22_O_4_ by HRESIMS data (*m*/*z* 301.1417 [M+Na]^+^, calcd. for C_16_H_22_O_4_Na, 301.1416). The 1D and 2D NMR data of **2** were similar but not identical to those of **1**, and by detailed analysis of HMBC and COSY data, the planar structure of **2** was determined to be the same as **1**, suggesting that **2** is a diastereomer of **1**. Additionally, the optical rotation values of **2** {${\left[\alpha \right]}_{D}^{25}$ +10.0 (*c* 0.1, MeOH)} and **1** {${\left[\alpha \right]}_{D}^{25}$ −20.0 (*c* 0.1, MeOH)} also supported their stereoisomeric relationship. 

The relative configuration of **2** was also determined by analysis of NOESY data and coupling constants. The strong NOESY correlations between H-4 and H-3, H-6 and H_3_-16 indicated that these protons were located on the same face of the molecule. The significant difference between **2** and **1** was the methyl group (H_3_-16) located on the same face with H-4 in **2**, while H-4 and H_3_-16 had a *trans*-relationship in **1**. Furthermore, the fact that H-4 and OH-14 had a co-facial relationship was determined by a similar procedure to that of **1**. Therefore, the relative configuration of **2** was determined as 2S*, 3R*, 4S*, 5R*, 14R* (**2A**) (Figure 3). 

**Figure 6:**
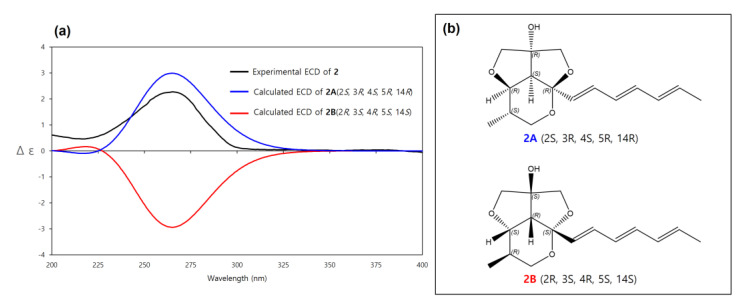
(**a**) Experimental and calculated ECD spectra of **2A** and **2B**. (**b**) Absolute configurations of **2A** (2*S*, 3*R*, 4*S*, 5*R*, 14*R*) and its enantiomer **2B** (2*R*, 3*S*, 4*R*, 5*S*, 14*S*).

The absolute configuration of **2** was determined by comparison of its experimental ECD spectrum with calculated ECD spectra of **2A** (2*S*, 3*R*, 4*S*, 5*R*, 14*R*) and its enantiomer **2B** (2*R*, 3*S*, 4*R*, 5*S*, 14*S*). The experimental ECD spectrum of **2** matched well with the calculated ECD spectrum of **2A** (Figure 6). Therefore, the absolute configuration of **2** was determined as 2*S*, 3*R*, 4*S*, 5*R*, 14*R*. Thus, **2** was a new diastereomer of **1** and named streptoglyceride F.

Compound **3** was isolated as a white powder. The molecular formula of **3** was determined to be C_17_H_24_O_4_ by HR-ESIMS data (*m/z* 315.1570 [M+Na]^+^, calcd. for C_17_H_24_O_4_Na 315.1572), one methylene group (-CH_2_-) more than that of **1**. The ^1^H and ^13^C NMR data of **3** were almost identical to that of **1**, except for the presence of one more methylene group at *δ*_C_ 21.5 (C-16) and *δ*_H_ 1.31 (H-16a) and 1.40 (H-16b). Furthermore, the continuous COSY correlations from H_3_-17 (*δ*_H_ 0.97) to H-2 (*δ*_H_ 1.69) via H-16a,b suggested that an ethyl group was substituted at C-2 in **3** instead of a methyl substitution in **1**. Thus, the planar structure of **3** was determined as 2-ethylstreptoglyceride B (Figure 2).

**Figure 7:**
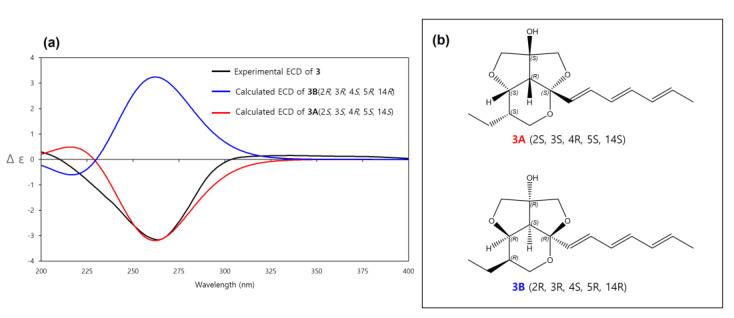
(**a**) Experimental and calculated ECD spectra of **3A** and **3B**. (**b**) Absolute configurations of **3A** (2*S*, 3*S*, 4*R*, 5*S*, 14*S*) and its enantiomer **3B** (2*R*, 3*R*, 4*S*, 5*R*, 14*R*).

The relative configuration of **3** was determined to be the same as **1** by the same afore-mentioned procedure for **1**, (2S*, 3S*, 4R*, 5S*, 14S*, (***3*A**)) (Figure 3), and the experimental ECD spectrum of **3** was compared to the calculated ECD spectra of **3A** and its enantiomer **3B**. The experimental ECD spectrum of **3** was well-matched to the calculated ECD spectrum of **3A** (Figure 7). Therefore, the absolute configuration of **3** was determined as 2*S*, 3*S*, 4*R*, 5*S*, 14*S*. Thus, the structure of **3** was determined as a new derivative of streptoglyceride B and named streptoglyceride G. 

Compound **4** was isolated as a white powder. The molecular formula of **4** was determined to be the same as that of **3**, C_17_H_24_O_4_ by HR-ESIMS data (*m/z* 315.1573 [M+Na]^+^, calcd. for C_17_H_24_O_4_Na, 315.1572). The 1D and 2D NMR data of **4** were also almost identical to those of **3** and by detailed analysis of HMBC and COSY data, the planar structure of **4** was determined to be the same as **3**, (2-ethylstreptoglyceride B). However, the optical rotation values of **4** {${\left[\alpha \right]}_{D}^{25}$ +30.0 (*c* 0.1, MeOH)} and **3** {${\left[\alpha \right]}_{D}^{25}$ −40.0 (*c* 0.1, MeOH)} suggested that they had a stereoisomeric relationship.

**Figure 8:**
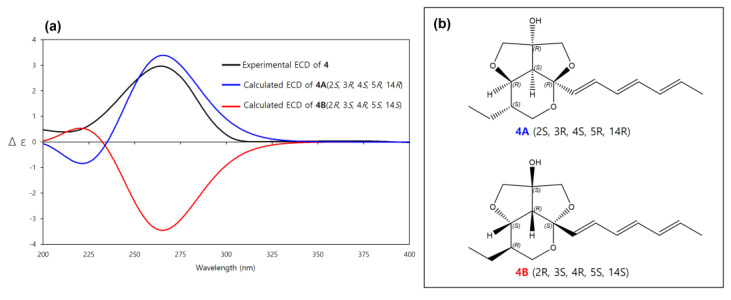
(**a**) Experimental and calculated ECD spectra of **4A** and **4B**. (**b**) Absolute configurations of **4A** (2*S*, 3*R*, 4*S*, 5*R*, 14*R*) and its enantiomer **4B** (2*R*, 3*S*, 4*R*, 5*S*, 14*S*).

The relative configuration of **4** was determined to be the same as **2** by the same above-mentioned procedure for **2** (2S*, 3R*, 4S*, 5R*, 14R*, (**4A**)) and the experimental ECD spectrum of **4** was compared to the calculated ECD spectra of **4A** (2*S*, 3*R*, 4*S*, 5*R*, 14*R*) and its enantiomer **4B** (2R, 3*S*, 4*R*, 5*S*, 14*S*). The experimental ECD spectrum of **4** was well matched to the calculated ECD spectrum of **4A** (Figure 8). Therefore, the absolute configuration of **4** was determined as 2*S*, 3*R*, 4*S*, 5*R*, 14*R*. Thus, **4** was determined as a new diastereomer of **3** and named streptoglyceride H.

**Scheme 1:**
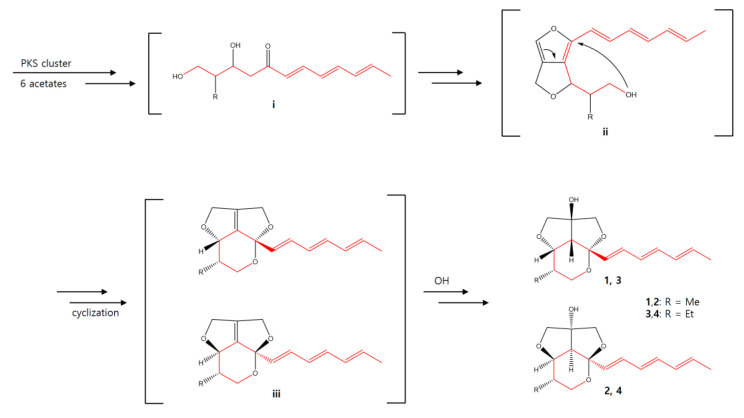
Plausible biosynthesis pathway of **1**–**4**.

The biosynthetic pathway of streptoglyceride A–D was previously proposed [20]. Similarly, we propose a plausible biosynthetic pathway of **1**–**4** (Scheme 1). The polyketide synthase (PKS) clusters would lead to the formation of a hexaketide intermediate **i** as a starting compound. The intermediate **i** could be converted to **ii** to form an intermediate with a rare 5/5 furo[3,4-*c*]furan ring. Afterward, cyclization of **ii** affords intermediate **iii**, which could be further converted into streptoglycerides E-H possessing a rare 6/5/5 ring system.

### 2.2. Bioactivities

Compounds **1**–**4** were screened for their effects on the production of NO in LPS-stimulated RAW 264.7 mouse macrophage cell line. All four compounds showed moderate inhibitory effects with IC_50_ values ranging from 3.5 to 10.9 μM (Table 2). The test was performed four times to confirm the reproducibility and statistical analyses were conducted using a t-test.

**Figure 9:**
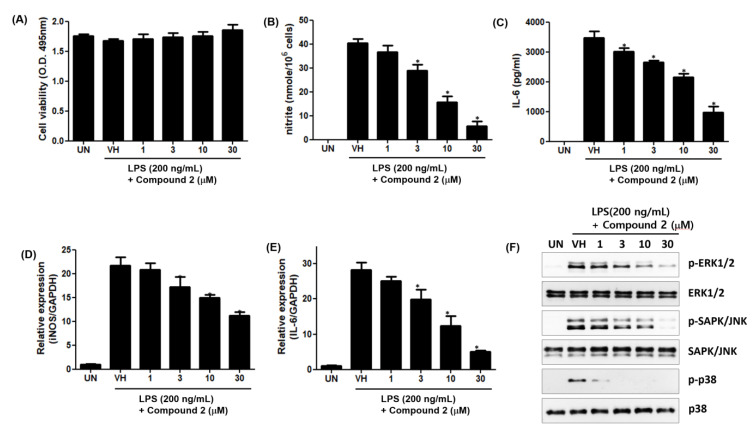
Inhibitory effects of **2** on LPS-induced nitrite production and IL-6 secretion in RAW 264.7 cells. RAW 264.7 cells were pretreated with the indicated concentrations of **2** for 1 h and stimulated with LPS (200 ng/mL) for 24 h. (**A**) Cell viability was determined by XTT assay. The levels of nitrite (**B**) and IL-6 (**C**) in culture supernatants were determined by Griess reaction and ELISA, respectively. The mRNA levels of iNOS (**D**) and IL-6 (**E**) were examined by qPCR. Data are represented as mean ± S.D. of quadruplicate determinations. * denotes that the response is significantly different from vehicle-treated group as determined by Dunnett’s multiple comparison test at *p* < 0.05. (**F**) MAPK activation was tested by Western Immunoblot Analysis. The results shown are representatives of more than two independent experiments.

To further investigate the anti-inflammatory effects of **2**, we examined the effect of **2** on LPS-induced production of inflammatory mediators, including NO and Interleukin-6 (IL-6), in RAW 264.7 cells. As shown in Figure 9A,B, the treatment of RAW 264.7 cells with LPS increased the accumulation of nitrite and IL-6, and **2** dose-dependently inhibited LPS-induced production of nitrite and IL-6 in LPS-stimulated RAW 264.7 cells. To further investigate whether the effects of **2** were due to its effects on the mRNA expression of cognate genes, we examined the effect of **2** on the mRNA expression of inducible nitric oxide synthase (iNOS) and IL-6 by qPCR. The mRNA levels of iNOS and IL-6 were induced by LPS treatment, and this induction was suppressed by **2** in a dose-dependent manner (Figure 9D,E). 

The concentrations of **2** used in this study had no cytotoxic effect on the viability of RAW 264.7 cells. Additionally, the mitogen-activated protein kinase (MAPK) activation study showed that phosphorylation of extracellular signal-regulated protein kinases (ERK), c-Jun N-terminal kinase (JNK) and p38 proteins was inhibited by **2** (Figure 9). The ERK, JNK and p38 proteins belonging to the MAPK superfamily are phosphorylated in the cytoplasm of stimulated cells by LPS. The activated p-ERK, p-JNK and p38 proteins activate transcription factors related with inflammation in their nucleus. Thus, **2** is considered to have anti-inflammatory activity by inhibiting the activation of the MAPK pathway.

## 3. Materials and Methods

### 3.1. General Experimental Procedures

The 1D and 2D NMR spectra were acquired on a Bruker 600 MHz spectrometer (Bruker BioSpin GmbH, Rheinstetten, Germany). UV-VIS spectra were acquired by a Shimadzu UV-1650PC spectrophotometer (Shimadzu Corporation, Kyoto, Japan). IR spectra were acquired on a JASCO FT/IR-4100 spectrophotometer (JASCO Corporation, Tokyo, Japan). Optical rotations were recorded on a Rudolph Research Analytical (Autopol III) polarimeter. High-resolution ESIMS experiments were performed on a hybrid ion-trap time-of-flight mass spectrometer (Shimadzu LC/MS-IT-TOF, Kyoto, Japan). HPLC was performed on a RI-101(Shodex). Semi-preparative HPLC was conducted using an ODS column (YMC-Pack-ODS-A, 250 × 10 mm i.d, 5 µm).

### 3.2. Isolation and Cultivation of the Strain 208DD-067 

The strain 208DD-067 was isolated from a sediment sample collected in Dokdo, South Korea in August 2020. The strain was identified as *Streptomyces specialis* on the basis of 16S rRNA gene sequence analysis (GenBank accession number OL691077). The seed and mass cultures of the strain 208DD-067 were performed in Bennett’s medium (BN broth, 1% glucose, 0.2% tryptone, 0.1% yeast extract, 0.1% beef extract, 0.5% glycerol, 3.2% sea salt, pH 7.0 before sterilization). A single colony of the strain from the agar plate was inoculated aseptically into a 2 L flask filled with 1 L of BN broth. After that, the strain was incubated at 24 °C for 7 days on a rotary shaker at 120 rpm and then the culture broth was transferred to a 100 L fermenter filled with 70 L of BN broth. The mass culture was incubated for 21 days at 28 °C and then harvested.

### 3.3. Extraction and Isolation of Metabolites

The culture broth (70 L) was centrifuged (60,000 rpm), and the supernatant was extracted twice with EtOAc (70 L). The EtOAc soluble part was evaporated to yield a crude extract (7.0 g). The extract was subjected to ODS open column chromatography followed by a stepwise gradient elution with MeOH in H_2_O (1:4, 2:3, 3:2, 4:1 and 100:0, *v/v*). The fraction eluted with 60% MeOH in H_2_O was purified by a semi-preparative reversed-phase HPLC (YMC-Pack-ODS-A, 250 × 10 mm i.d, 5 *µ*m, flow rate 2.0 mL/min, RI detector) using an isocratic elution with 60% MeOH in H_2_O to yield **1** (0.5 mg, *t*_R_ = 36 min) and **2** (0.9 mg, *t*_R_ = 31 min) and the fraction eluted with 80% MeOH in H_2_O was purified by a semi-preparative reversed-phase HPLC (YMC-Pack-ODS-A, 250 × 10 mm i.d, 5 *µ*m, flow rate 2.0 mL/min, RI detector) using an isocratic elution with 50% ACN in H_2_O to yield **3** (0.9 mg, *t*_R_ = 20 min) and **4** (1.2 mg, *t*_R_ = 15 min).

Streptoglyceride E (**1**): a white powder; ${\left[\alpha \right]}_{D}^{25}$ −20.0 (*c* 0.1, MeOH); IR *ν*_max_ 3343, 2960, 2840, 1643, 1465, 1409, 1014 UV (MeOH) λ_max_ (log ε) 202 (3.45), 266 (2.48) nm; HRESIMS *m/z* 301.1417 [M+Na]^+^ (calcd for C_16_H_22_O_4_Na, 301.1416); ^1^H and ^13^C NMR data (CD_3_OD, 600 MHz and 150 MHz, respectively), Table 1.

Streptoglyceride F (**2**): a white powder; ${\left[\alpha \right]}_{D}^{25}$ +10.0 (*c* 0.1, MeOH); IR *ν*_max_ 3386, 2921, 2864, 2364, 2314, 1597, 1412, 1057 UV (MeOH) λ_max_ (log ε) 266 (2.45) nm; HRESIMS *m/z* 301.1417 [M+Na]^+^ (calcd for C_16_H_22_O_4_Na, 301.1416); ^1^H and ^13^C NMR data (CD_3_OD, 600 MHz and 150 MHz, respectively), Table 1.

Streptoglyceride G (**3**): a white powder; ${\left[\alpha \right]}_{D}^{25}$ −40.0 (*c* 0.1, MeOH); IR *ν*_max_ 3356, 2967, 2349, 2311, 1649, 1543, 1066 UV (MeOH) λ_max_ (log ε) 231 (2.45), 265 (2.48) nm; HRESIMS *m/z* 315.1570 [M+Na]^+^ (calcd for C_17_H_24_O_4_Na, 315.1572); ^1^H and ^13^C NMR data (CD_3_OD, 600 MHz and 150 MHz, respectively), Table 1.

Streptoglyceride H (**4**): a white powder; ${\left[\alpha \right]}_{D}^{25}$ +30.0 (*c* 0.1, MeOH); IR *ν*_max_ 3392, 2965, 2929, 2876, 1646, 1596, 1455, 1374, 1212, 1059 UV (MeOH) λ_max_ (log ε) 266 (2.45) nm; HRESIMS *m/z* 315.1573 [M+Na]^+^ (calcd for C_17_H_24_O_4_Na, 315.1572); ^1^H and ^13^C NMR data (CD_3_OD, 600 MHz and 150 MHz, respectively), Table 1.

### 3.4. Cell Viability Assay

Cell viability assay was performed using a Cell Proliferation Kit II (Roche Applied Science, Mannheim, Germany) according to the manufacturer’s instructions. Briefly, the XTT labeling mixture was prepared by mixing 50 volumes of 1 mg/mL sodium 3’-[1-(phenylaminocarbonyl)-3,4-tetrazolium]-bis(4-methoxy-6-nitro)benzene sulfonic acid hydrate with 1 volume of 0.383 mg/ml of N-methyldibenzopyrazine methyl sulfate, added to the cultures and incubated for 2 h at 37 °C. Absorbance was measured at 495 nm with a reference wavelength at 650 nm.

### 3.5. Computational Methods

Conformational analysis was performed using molecular mechanics force-field (MMFF94s) calculations with a search limit of 3.0 kcal mol^−1^ in CONFLEX version 8.0 program (CONFLEX Corporation, Tokyo, Japan). The low-energy conformers were further optimized using the density functional theory (DFT) method at the B3LYP/6-311G+(d,p) level. The theoretical ECD spectra were calculated by Gaussian 16 software (Gaussian Inc., Wallingford, CT, USA) using the time-dependent density functional theory (TD-DFT) method at the B3LYP/6-311+G (d,p) level in MeOH with the Polarizable Continuum Model (PCM) utilizing the integral equation formalism variant (IEFPCM) model. The Boltzmann distributions of conformers were calculated based on their Hartrees energy, and the calculated ECD spectra were averaged according to the Boltzmann distributions. NMR chemical shifts were calculated by a gauge-independent atomic orbitals (GIAO) method at the B3LYP/6-311+G(d,P)/IEFPCM level in MeOH.

### 3.6. Nitrite Quantification

NO_2_^−^ accumulation was used as an indicator of NO production in the medium. RAW 264.7 cells were plated at 5 × 10^5^ cells/mL and stimulated with LPS (200 ng/mL) in the presence or absence of **2** (1, 3, 10 or 30 μM) for 24 h. The isolated supernatants were mixed with an equal volume of Griess reagent (1% sulfanilamide, 0.1% naphthylethylenediamine dihydrochloride, and 2% phosphoric acid), and incubated at room temperature for 10 min. NaNO_2_ was used to generate a standard curve, and nitrite production was determined by measuring the optical density at 540 nm. 

### 3.7. Enzyme-Linked Immunosorbent Assay (ELISA)

RAW 264.7 cells were plated at 5 × 10^5^ cells/mL and stimulated with LPS (200 ng/mL) in the presence or absence of compound 2 (1, 3, 10 or 30 μM) for 24 h. The culture supernatants were collected, and the amount of IL-6 was determined by mouse IL-6 ELISA kit (R&D Systems, Minneapolis, MN, USA) according to the manufacturer’s instructions.

### 3.8. RNA Isolation and Quantification of mRNA Expression by qPCR

Total cellular RNA was extracted using an RNeasy Plus Mini Kit (Qiagen, Valencia, CA, USA) with RNase-Free DNase Set (Qiagen) according to the manufacturer’s instructions. cDNA was generated from total RNA by reverse transcription using AccuPower RT PreMix (Bioneer). The resulted cDNA was amplified by qPCR in conjunction with Power SYBR Green PCR Master Mix (Invitrogen, Carlsbad, CA, USA). For qPCR, samples were amplified by 45 cycles of denaturation (95 °C for 15s) and amplification (60 °C for 1 min using ABI 7500 Sequence Detection System (Applied Biosciences, Foster City, CA, USA). The gene expression levels relative to control gene (GAPDH) was calculated by 2^−^^ΔΔ^^Ct^ method.

### 3.9. Western Immunoblot Analysis

Twenty micrograms of whole-cell lysates were separated by 10% sodium dodecyl sulfate-polyacrylamide gel electrophoresis and electro-transferred to a nitrocellulose membrane (Amersham Biosciences UK, Ltd., Little Chalfont, Buckinghamshire, UK). Each membrane was pre-incubated for 1 h at room temperature in Tris-buffered saline, pH 7.6, containing 0.05% Tween 20 and 5% nonfat milk. Each nitrocellulose membrane was incubated with specific antibodies against p-ERK1/2, ERK1/2, p-SAPK/JNK, SAPK/JNK, p-p38 and p38 (Cell Signaling Technology, Danvers, MA, USA). Immunoreactive bands were then detected by incubating with secondary IgG antibody conjugated with horseradish peroxidase and visualizing with enhanced chemiluminescence reagents (GE Healthcare, Chicago, IL, USA). 

### 3.10. Statistical Analysis

One-way ANOVA followed by Dunnett’s multiple comparison test was used for statistical analysis using GraphPad Prism (GraphPad Software, La Jolla, CA, USA). The criteria for statistical significance were set at * *p* < 0.05.

## 4. Conclusions

We isolated four new streptoglycerides E–H (**1**–**4**) from a marine-derived actinomycete *Streptomyces specialis*. The structures of the new compounds (**1**–**4**) were elucidated by detailed analysis of HR-ESI-MS, 1D and 2D NMR data and ECD calculations. All compounds showed good anti-inflammatory activity with IC_50_ values ranging from 3.5 to 10.9 µM. Furthermore, the results demonstrated that **2** inhibited the mRNA expression of iNOS and IL-6 without cytotoxicity in RAW264.7 cells. According to this study, **2** inhibited the phosphorylation of ERK, JNK and p38 proteins in the MAPK pathway. To the best of our knowledge, this study represents the third example of marine natural products with a rare 6/5/5/-membered ring system and has anti-inflammatory activity. Streptoglycerides E–H (**1**–**4**) are a promising series of compounds with good anti-inflammatory activity and the potential for further therapeutic lead identification.

## Figures and Tables

**Table 1:** ^1^H and ^13^C NMR data for **1**–**4** at 600 MHz and 150 MHz in CD_3_OD (*δ* in ppm, *J* in Hz), respectively.

No	1		2		3		4	
	*δ*_H_, mult (*J* in Hz)	*δ*_C_	*δ*_H_, mult (*J* in Hz)	*δ*_C_	*δ*_H_, mult (*J* in Hz)	*δ*_C_	*δ*_H_, mult (*J* in Hz)	*δ*_C_
1a	3.36, dd (10.9, 4.3)	62.3	3.35, dd (11.2, 1.4)	61.7	3.44, dd (10.9, 4.3)	61.3	3.49, d-like	59.3
1b	3.74, overlapped		4.13, dd (11.2, 2.1)		3.75, overlapped		4.08, overlapped	
2	1.92, m	32.1	1.80, m	31.1	1.69, m	38.8	1.52, m	38.2
3	4.11, dd (7.0, 3.1)	80.0	3.99, overlapped	81.4	4.21, dd (7.1, 3.1)	78.2	4.07, overlapped	80.4
4	2.41, d (7.0)	54.3	2.40, d (7.2)	51.5	2.41, d (7.1)	54.3	2.38, d (7.3)	51.8
5		106.1		106.2		106.4		106.2
6	5.66, d (15.3)	133.6	5.68, d (15.3)	133.7	5.67, d (15.4)	133.6	5.67, d (15.3)	133.7
7	6.40, dd (15.3, 10.6)	131.8	6.41, dd (15.3, 10.8)	131.5	6.41, dd (15.3, 10.7)	131.8	6.39, dd (15.3, 10.6)	131.5
8	6.11, overlapped	130.3	6.12, overlapped	130.2	6.12, overlapped	130.3	6.12, overlapped	130.2
9	6.26, dd (14.9, 10.6)	136.0	6.27, dd (14.9, 10.5)	136.1	6.26, dd (14.9, 10.6)	136.0	6.26, dd (14.9, 10.6)	136.1
10	6.10, overlapped	132.9	6.11, overlapped	132.9	6.11, overlapped	132.9	6.11, overlapped	132.9
11	5.75, m	131.5	5.76, m	131.5	5.76, m	131.5	5.76, m	131.5
12	1.76, d (7.0)	18.4	1.76, d (6.1)	18.4	1.77, d (6.6)	18.4	1.76, d (7.0)	18.4
13a	3.62, overlapped	79.7	3.64, d (8.8)	79.3	3.62, d (8.9)	79.7	3.64, d (8.9)	79.2
13b	4.02, overlapped		4.01, overlapped		4.01, overlapped		4.03, overlapped	
14		87.4		88.4		88.0		88.5
15a	3.76, overlapped	78.4	3.77, d (9.3)	78.2	3.77, overlapped	78.4	3.78, d (9.3)	78.3
15b	4.00, overlapped		4.00, overlapped		4.00, overlapped		4.02, overlapped	
16a	0.94, d (7.0)	13.0	1.07, d (7.3)	15.2	1.31, m	21.5	1.42, m	22.9
16b					1.40, m		1.48, m	
17					0.97, t (7.4)	11.5	1.02, t (7.3)	12.1

**Table 2:** Inhibitory effects of **1**–**4** on LPS-induced NO production in RAW 264.7 cells.

Compounds	IC_50_, μM
1	10.9
2	5.9
3	4.7
4	3.5

IC_50_ values are the concentration corresponding to 50% inhibition of NO production.

## Data Availability

The data presented in the article are available in the Supplementary Materials.

## Supplementary Materials

The following are available online at https://www.mdpi.com/article/10.3390/md20010044/s1, Figures S1–S41: The analyzed data of MS, IR, 1D and 2D NMR spectra and Energy-minimized models of conformers of compounds **1**–**4**, Table S1–S9: The calculated ECD data of compounds **1**–**4**.
