# Supplementary material for: Streptoglycerides E–H, Unsaturated Polyketides from the Marine-Derived Bacterium Streptomyces specialis and Their Anti-Inflammatory Activity
Source: Mar Drugs. 2022 Jan 1;20(1):44. doi: 10.3390/md20010044 (PMC8781396; doi:10.3390/md20010044)
Supplement: Supplementary file 1 [file marinedrugs-20-00044-s001.zip › marinedrugs-1522455-supplementary.pdf]

marine drugs

MDPI

Streptoglycerides E–H, Unsaturated Polyketides from the Marine-Derived Bacterium *Streptomyces specialis* and Their Anti-Inflammatory Activity

Hee Jae Shin [1,2,*], Chang-Su Heo [1,2], Cao Van Anh [1,2], Yeo Dae Yoon [3] and Jong Soon Kang [3]

1 Marine Natural Products Chemistry Laboratory, Korea Institute of Ocean Science and Technology, 385 Haeyang-ro, Yeongdo-gu, Busan 49111, Korea; science30@kiost.ac.kr (C.-S.H.); caovananh@kiost.ac.kr (C.V.A.)
2 Department of Marine Biotechnology, University of Science and Technology (UST), 217 Gajungro, Yuseong-gu, Daejeon 34113, Korea
3 Laboratory Animal Resource Center, Korea Research Institute of Bioscience and Biotechnology, 30 Yeongudanjiro, Cheongju 28116, Korea; kanjon@kribb.re.kr (J.S.K.); yunyd76@kribb.re.kr (Y.D.Y.)
* Correspondence: shinhj@kiost.ac.kr; Tel.: +82-51-664-3341; Fax: +82-51-664-3340

Contents

Figure S1. HRESIMS data of **1**. 3
Figure S2. IR spectrum of **1**. 4
Figure S3. ^{1}H NMR data of **1** (600 MHz, CD_3OD). 4
Figure S4. ^{13}C NMR data of **1** (150 MHz, CD_3OD). 4
Figure S5. HSQC spectrum of **1** (CD_3OD). 5
Figure S6. COSY spectrum of **1** (CD_3OD). 5
Figure S7. HMBC spectrum of **1** (CD_3OD). 6
Figure S8. NOESY spectrum of **1** (CD_3OD). 6
Figure S9. HRESIMS data of **2**. 7
Figure S10. IR spectrum of **2**. 8
Figure S11. ^{1}H NMR data of **2** (600 MHz, CD_3OD). 8
Figure S12. ^{13}C NMR data of **2** (150 MHz, CD_3OD). 8
Figure S13. HSQC spectrum of **2** (CD_3OD). 9
Figure S14. COSY spectrum of **2** (CD_3OD). 9
Figure S15. HMBC spectrum of **2** (CD_3OD). 10
Figure S16. NOESY spectrum of **2** (CD_3OD). 10
Figure S17. HRESIMS data of **3**. 11
Figure S18. IR spectrum of **3**. 12
Figure S19. ^{1}H NMR data of **3** (600 MHz, CD_3OD). 12
Figure S20. ^{13}C NMR data of **3** (150 MHz, CD_3OD). 12
Figure S21. HSQC spectrum of **3** (CD_3OD). 13
Figure S22. COSY spectrum of **3** (CD_3OD). 13
Figure S23. HMBC spectrum of **3** (CD_3OD). 14
Figure S24. NOESY spectrum of **3** (CD_3OD). 14
Figure S25. HR-ESIMS data of **4**. 15
Figure S26. IR spectrum of **4**. 16
Figure S27. ^{1}H NMR data of **4** (600 MHz, CD_3OD). 16
Figure S28. ^{13}C NMR data of **4** (150 MHz, CD_3OD). 16
Figure S29. HSQC spectrum of **4** (CD_3OD). 17
Figure S30. COSY spectrum of **4** (CD_3OD). 17
Figure S31. HMBC spectrum of **4** (CD_3OD). 18
Figure S32. NOESY spectrum of **4** (CD_3OD). 18
Table S1. Experimental chemical shifts and calculated chemical shifts of **1** and **1′** for DP4+ calculation. 19
Figure S33. Result sheet of DP4+ probability calculations of isomers of **1**. 20
Figure S34. Energy-minimized models of conformers of **1A**. 21
Table S2. Hartrees energy, and Boltzmann's distribution of conformers of **1A**. 21
Figure S35. Energy-minimized models of conformers of **1B**. 22
Table S3. Hartrees energy, and Boltzmann's distribution of conformers of **1B**. 22
Figure S36. Energy-minimized models of conformers of **2A**. 23
Table S4. Hartrees energy, and Boltzmann's distribution of conformers of **2A**. 23
Figure S37. Energy-minimized models of conformers of **2B**. 24
Table S5. Hartrees energy, and Boltzmann's distribution of conformers of **2B**. 24
Figure S38. Energy-minimized models of conformers of **3A**. 25
Table S6. Hartrees energy, and Boltzmann's distribution of conformers of **3A**. 26
Figure S39. Energy-minimized models of conformers of **3B**. 27
Table S7. Hartrees energy, and Boltzmann's distribution of conformers of **3B**. 28
Figure S40. Energy-minimized models of conformers of **4A**. 29
Table S8. Hartrees energy, and Boltzmann's distribution of conformers of **4A**. 29

Figure S41. Energy-minimized models of conformers of **4B**. 30
Table S9. Hartrees energy, and Boltzmann's distribution of conformers of **4B**. 30

Elemental Composition Report

Single Mass Analysis
Tolerance = 5.0 PPM / DBE: min = -1.5, max = 100.0
Element prediction: Off
Number of isotope peaks used for i-FIT = 3

Monoisotopic Mass, Even Electron Ions
41 formula(e) evaluated with 1 results within limits (all results (up to 1000) for each mass)
Elements Used:
C: 0-20 H: 0-30 O: 0-5 Na: 0-1

Minimum:				-1.5				
Maximum:		100.0	5.0	100.0				
Mass	Calc. Mass	mDa	PPM	DBE	i-FIT	Norm	Conf(%)	Formula
301.1417	301.1416	0.1	0.3	5.5	970.4	n/a	n/a	C16 H22 O4 Na

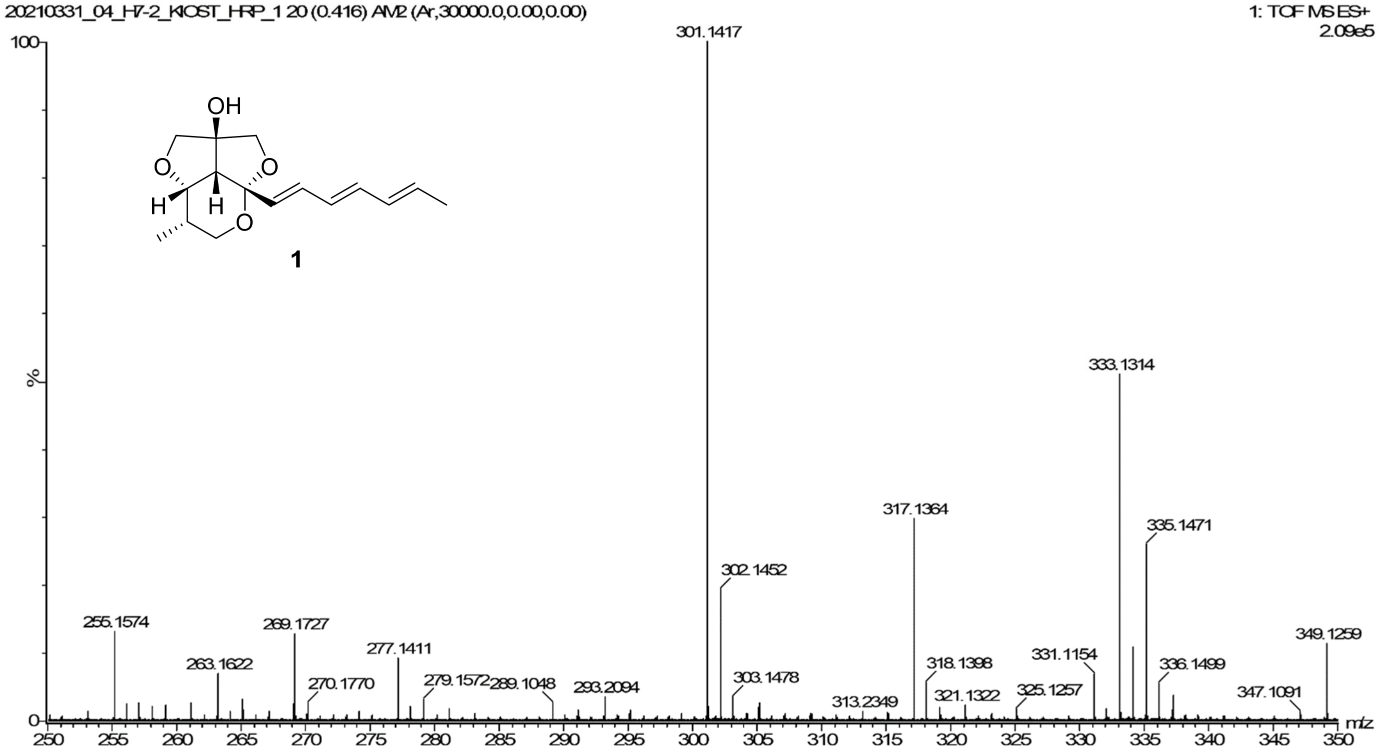

Figure S1. HRESIMS data of **1**.

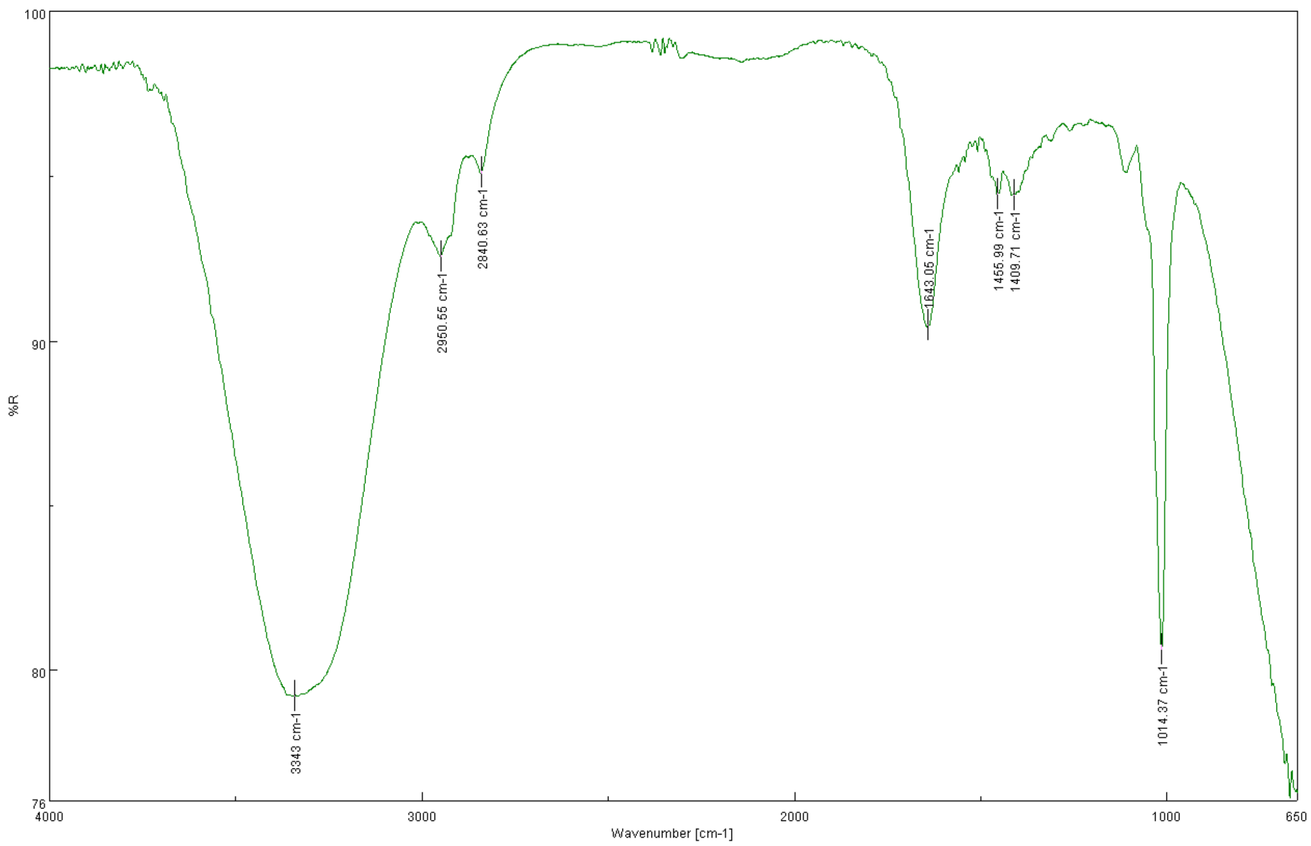

Figure S2. IR spectrum of **1**.

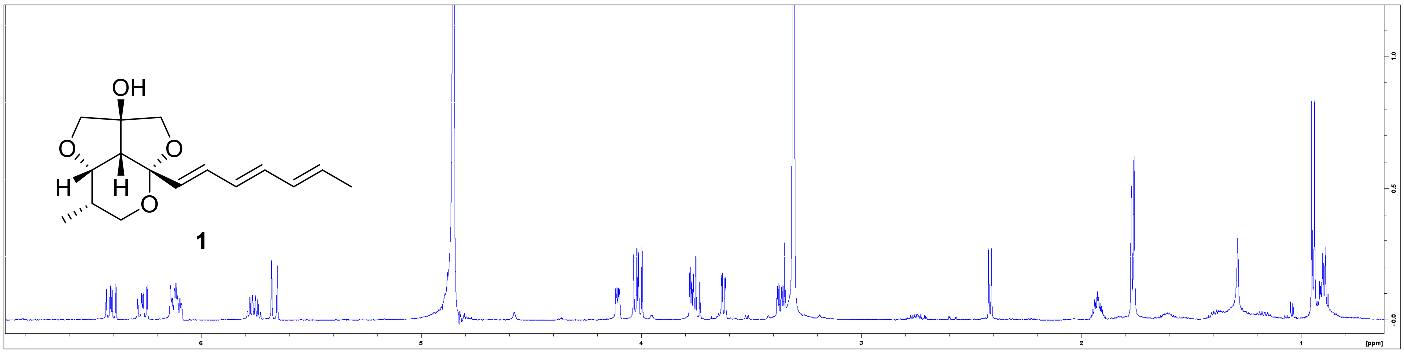

Figure S3. ^{1}H NMR spectrum of streptoglyceride E (**1**) in CD_3OD (600MHz).

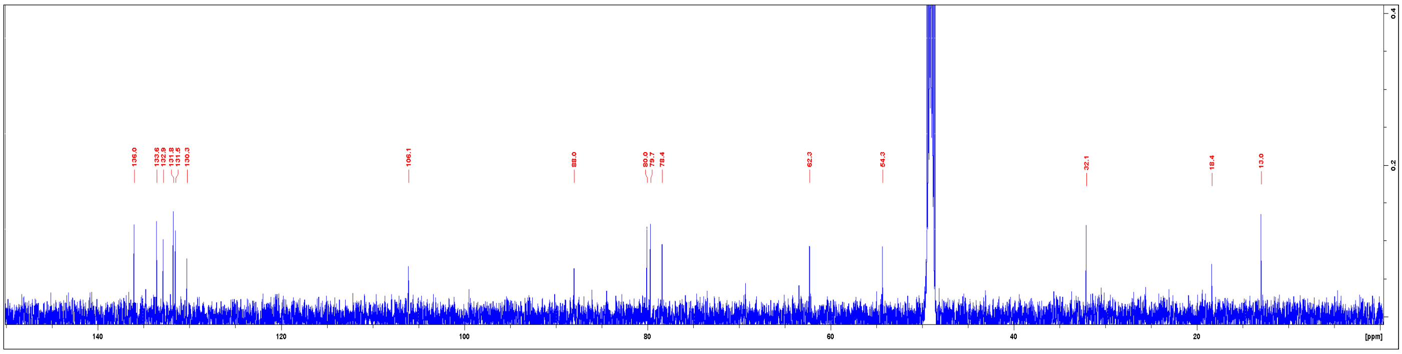

Figure S4. ^{13}C NMR spectrum of streptoglyceride E (**1**) in CD_3OD (150MHz).

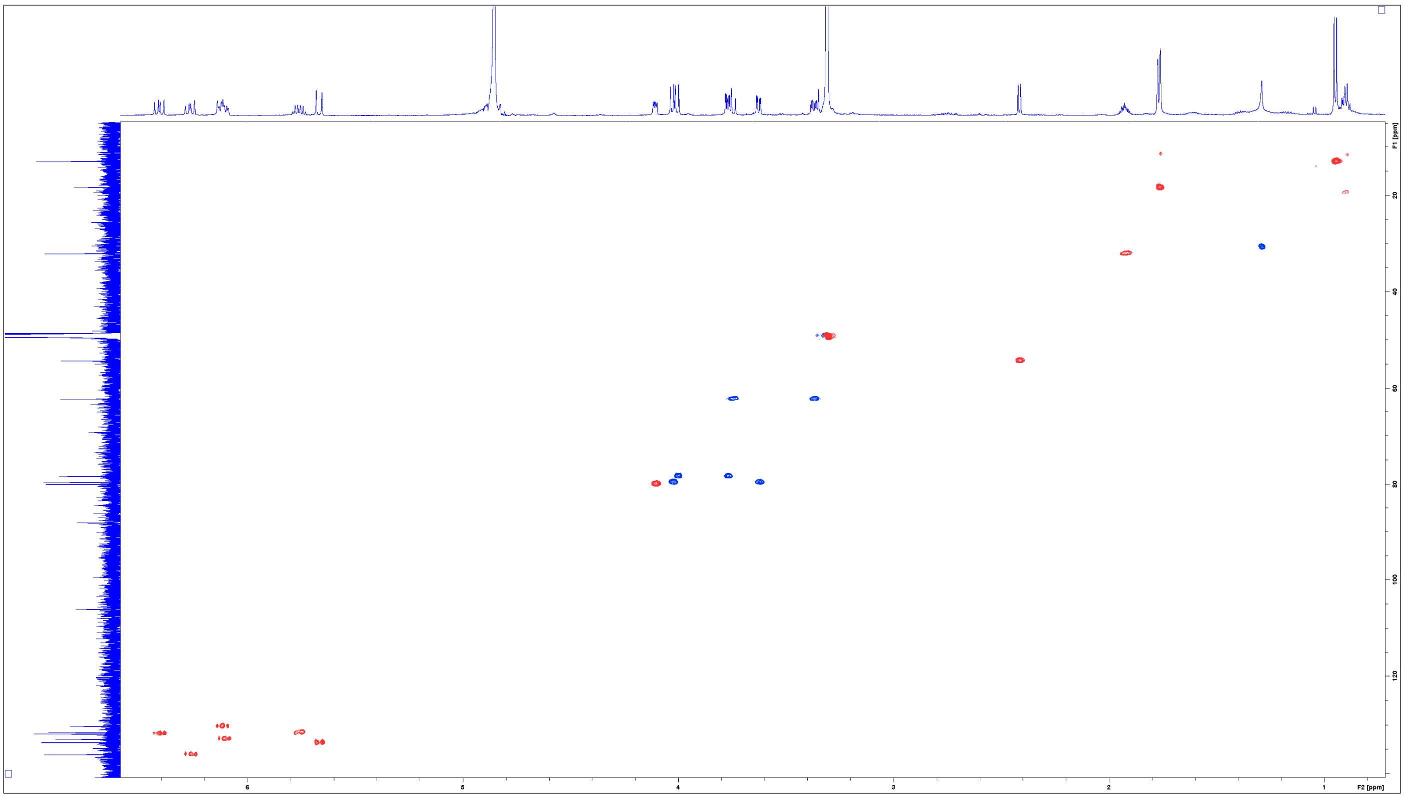

Figure S5. HSQC spectrum of streptoglyceride E (**1**) in CD_3OD (^1H: 600MHz, ^{13}C: 150MHz).

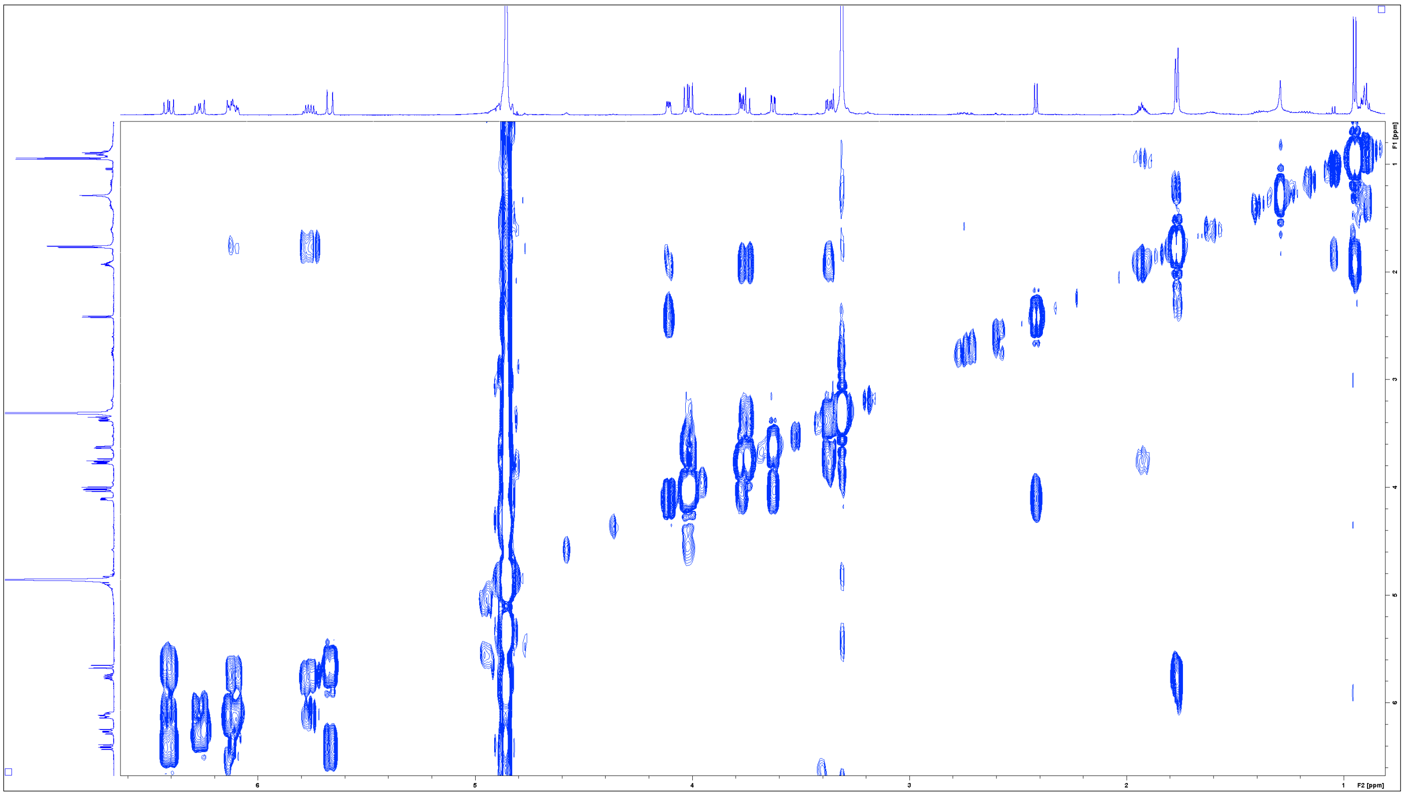

Figure S6. COSY spectrum of streptoglyceride E (**1**) in CD3OD (^{1}H: 600MHz).

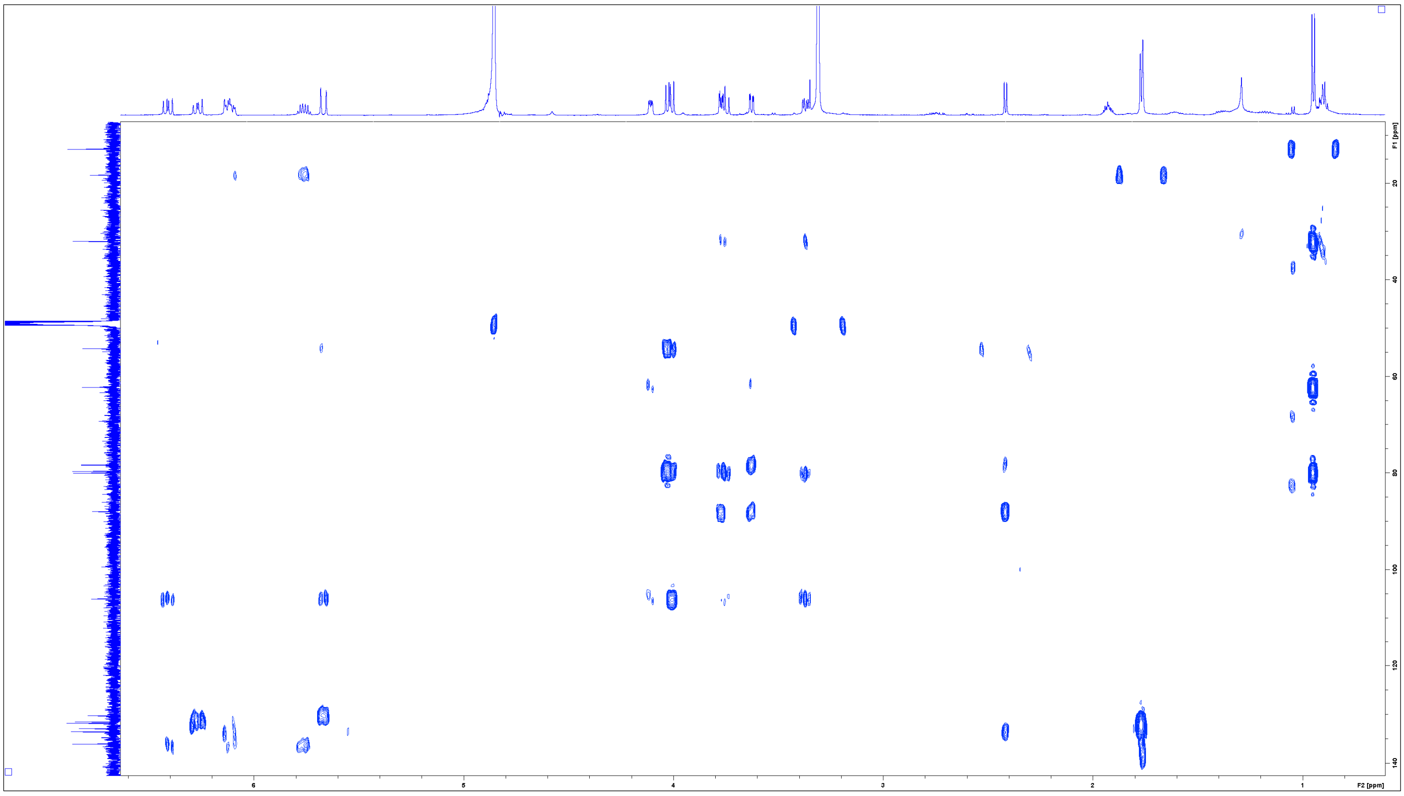

Figure S7. HMBC spectrum of streptoglyceride E (**1**) in CD3OD (^{1}H: 600MHz, ^{13}C: 150MHz).

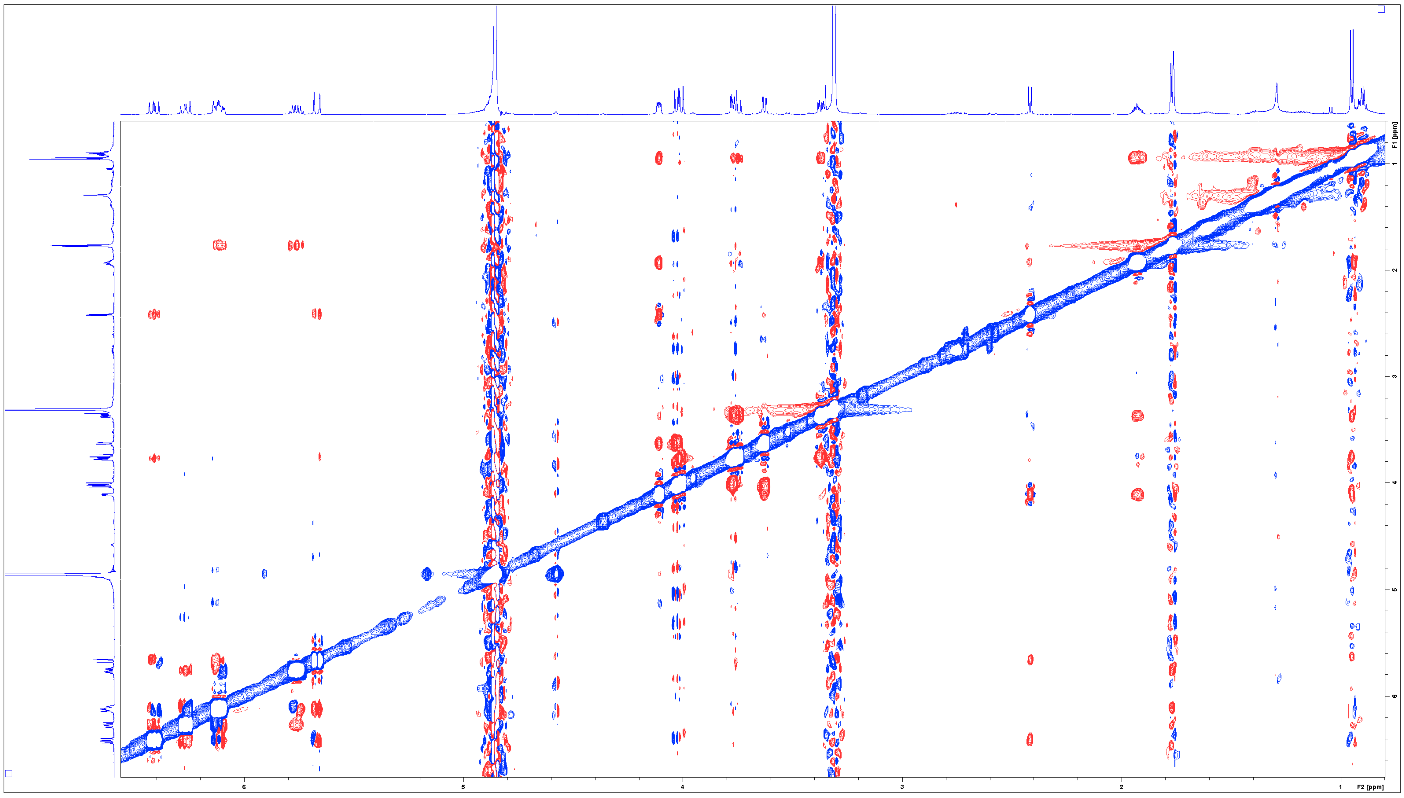

Figure S8. NOESY spectrum of streptoglyceride E (**1**) in CD_3OD (^{1}H: 600MHz).

Elemental Composition Report

Single Mass Analysis
Tolerance = 5.0 PPM / DBE: min = -1.5, max = 100.0
Element prediction: Off
Number of isotope peaks used for i-FIT = 3

Monoisotopic Mass, Even Electron Ions
41 formula(e) evaluated with 1 results within limits (all results (up to 1000) for each mass)
Elements Used:
C: 0-20 H: 0-30 O: 0-5 Na: 0-1

Minimum:				-1.5				
Maximum:		100.0	5.0	100.0				
Mass	Calc. Mass	mDa	PPM	DBE	i-FIT	Norm	Conf(%)	Formula
301.1417	301.1416	0.1	0.3	5.5	973.0	n/a	n/a	C16 H22 O4 Na

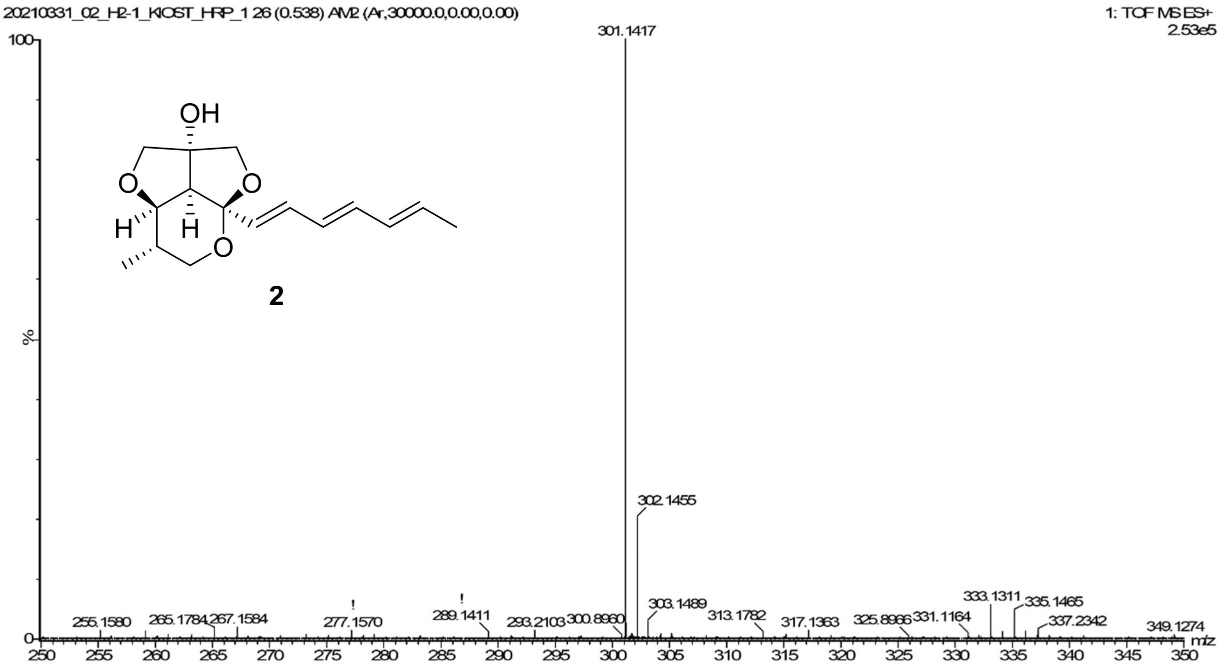

Figure S9. HRESIMS data of **2**.

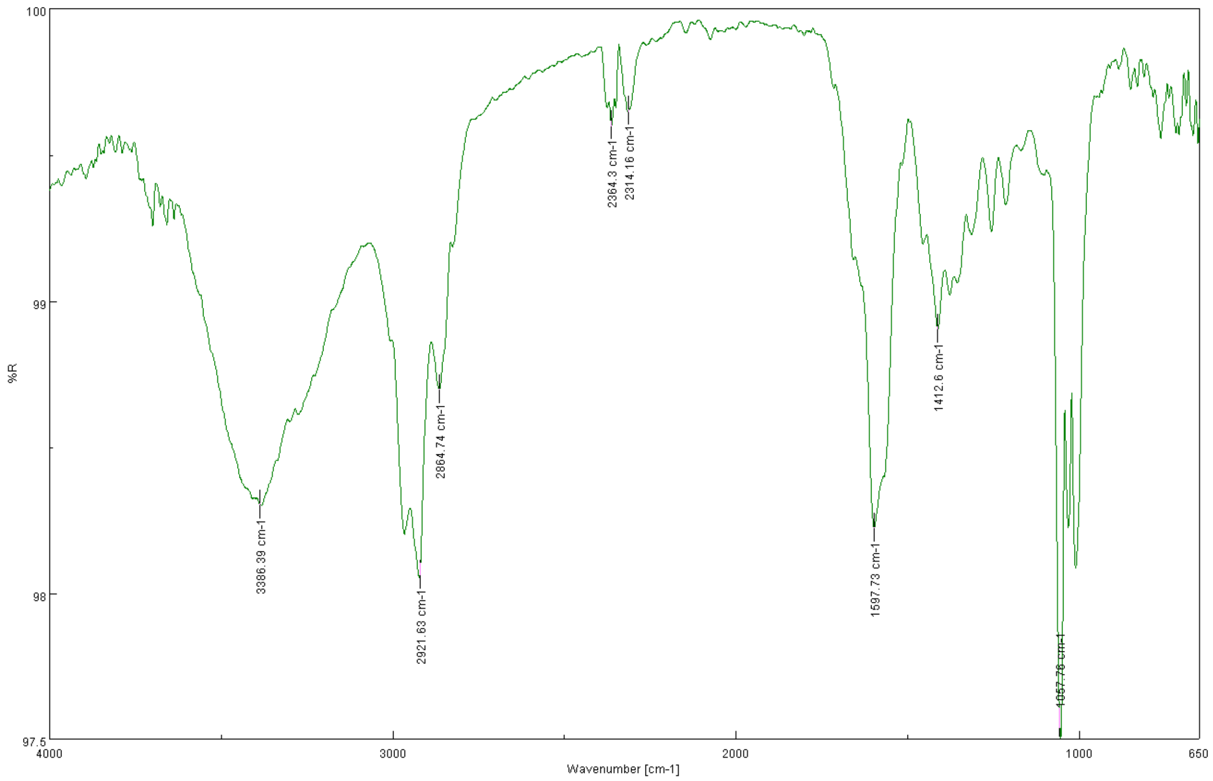

Figure S10. IR spectrum of **2**.

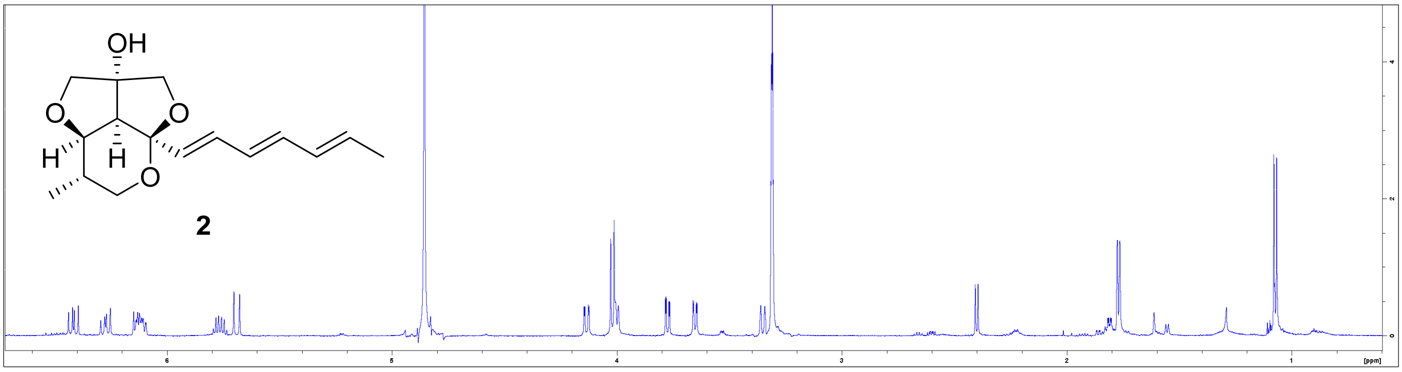

Figure S11. ^{1}H NMR spectrum of streptoglyceride F (**2**) in CD_3OD (600MHz).

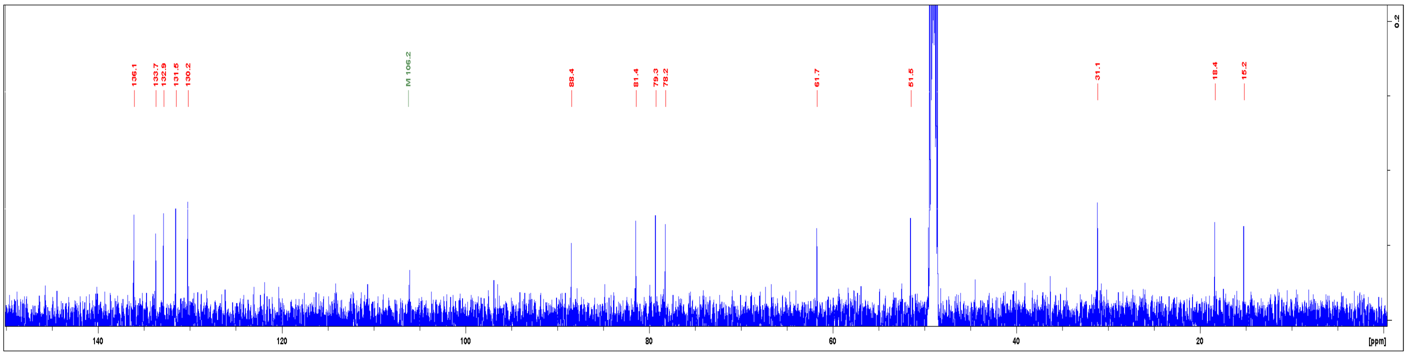

Figure S12. ^{13}C NMR spectrum of streptoglyceride F (2) in CD_3OD (150MHz).

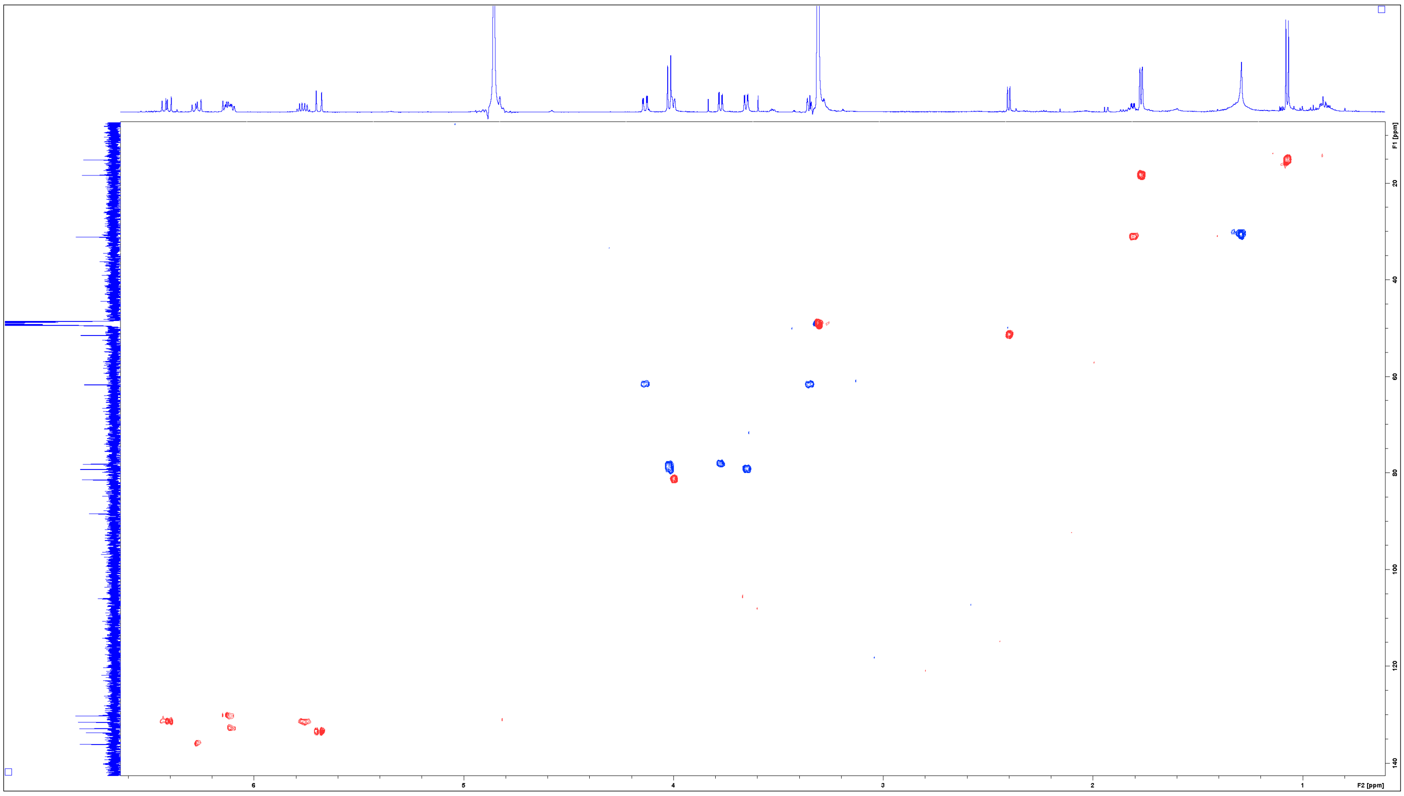

Figure S13. HSQC spectrum of streptoglyceride F (**2**) in CD_3OD (^1H: 600MHz, ^{13}C: 150MHz).

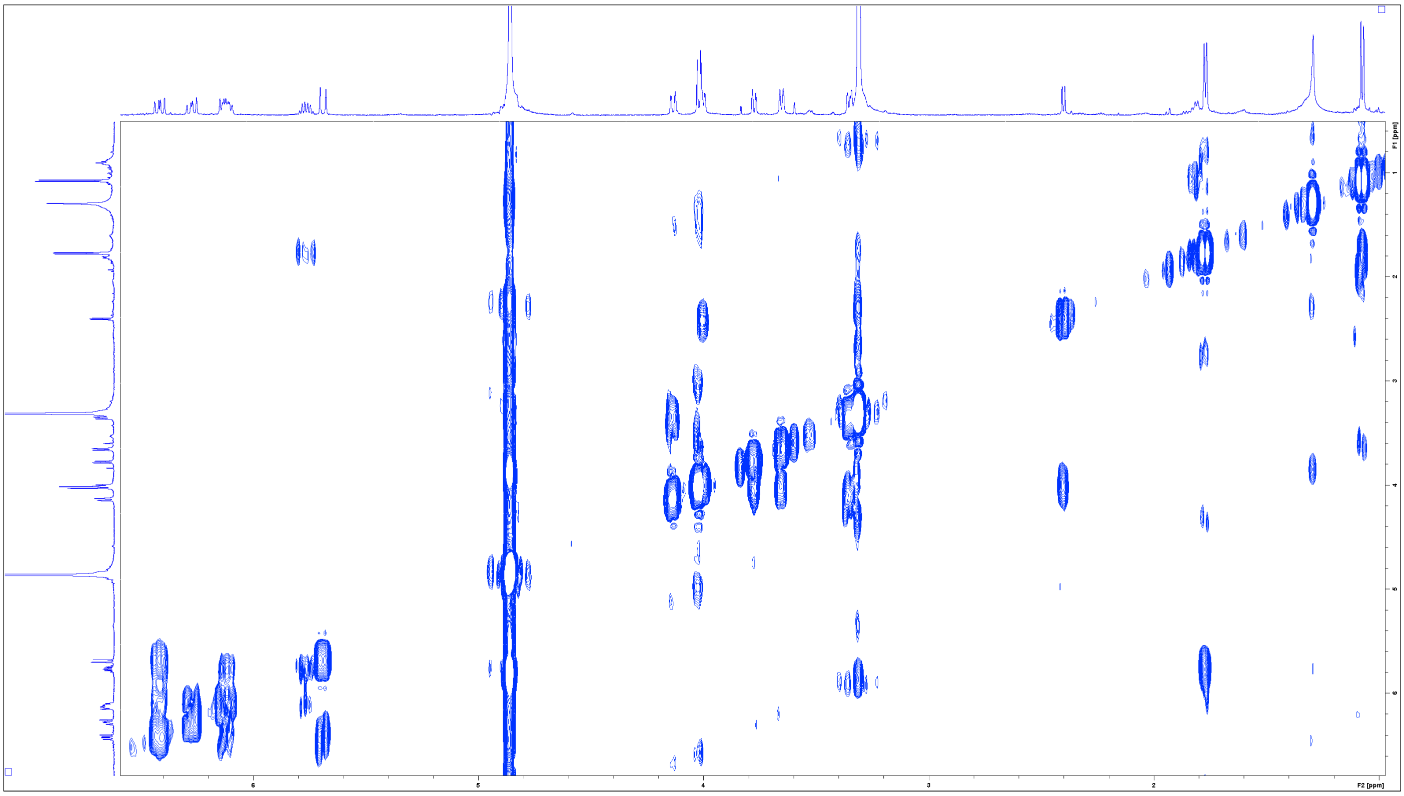

Figure S14. COSY spectrum of streptoglyceride F (**2**) in CD_3OD (^1H: 600MHz).

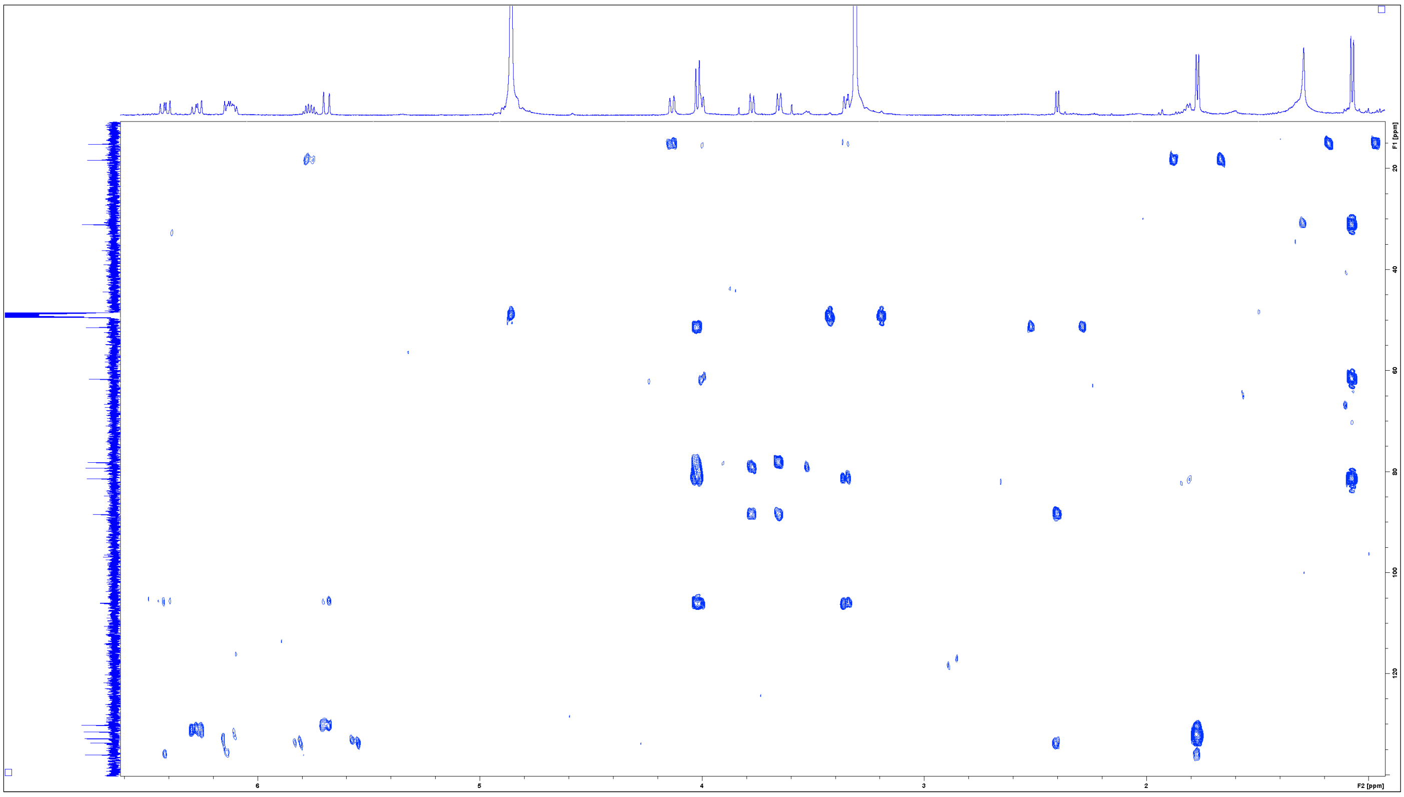

Figure S15. HMBC spectrum of streptoglyceride F (**2**) in CD3OD (^{1}H: 600MHz, ^{13}C: 150MHz).

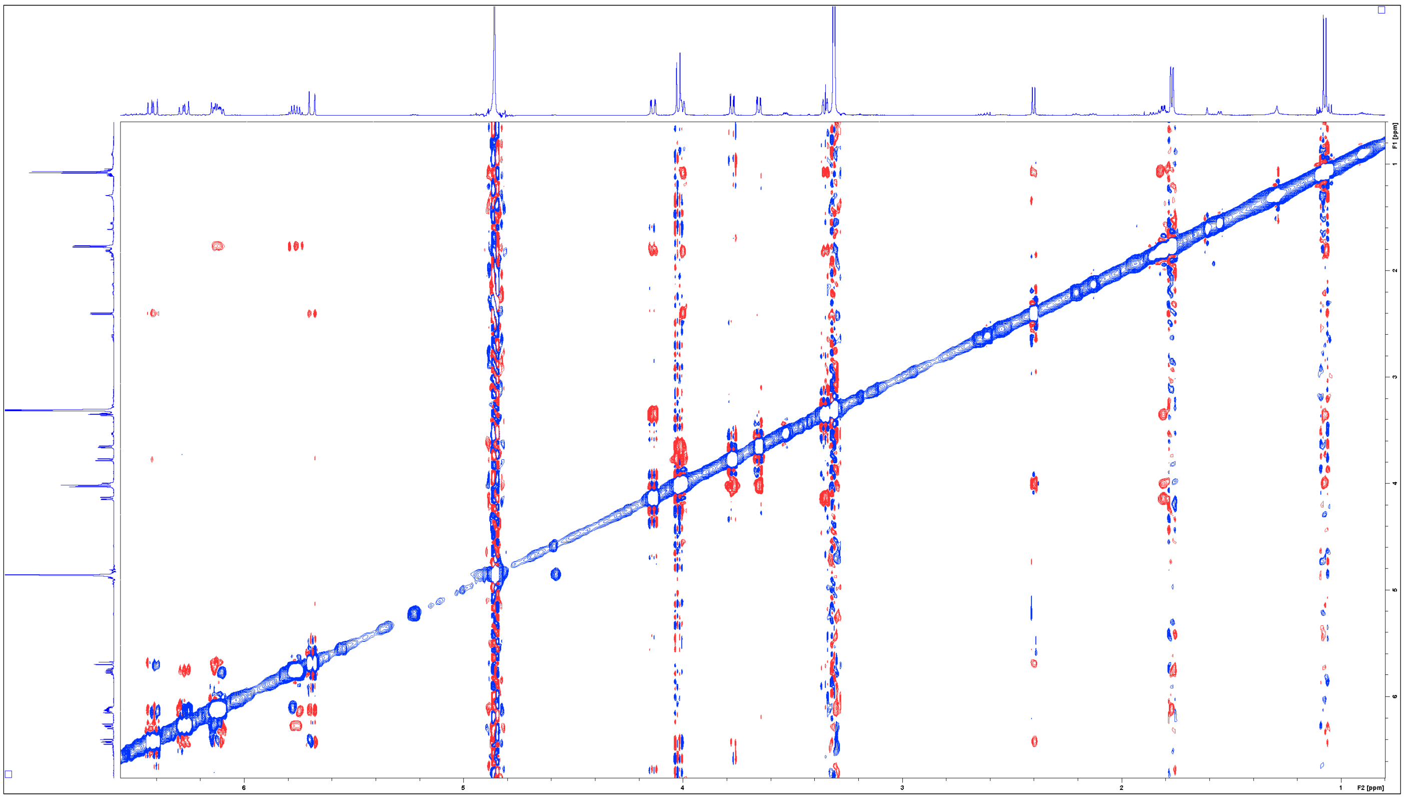

Figure S16. NOESY spectrum of streptoglyceride F (**2**) in CD3OD (^{1}H: 600MHz).

Elemental Composition Report

Single Mass Analysis
Tolerance = 5.0 PPM / DBE: min = -1.5, max = 100.0
Element prediction: Off
Number of isotope peaks used for i-FIT = 3

Monoisotopic Mass, Even Electron Ions
39 formula(e) evaluated with 1 results within limits (all results (up to 1000) for each mass)
Elements Used:
C: 0-20 H: 0-30 O: 0-5 Na: 0-1

Minimum:				-1.5				
Maximum:		100.0	5.0	100.0				
Mass	Calc. Mass	mDa	PPM	DBE	i-FIT	Norm	Conf(%)	Formula
315.1570	315.1572	-0.2	-0.6	5.5	987.5	n/a	n/a	C17 H24 O4 Na

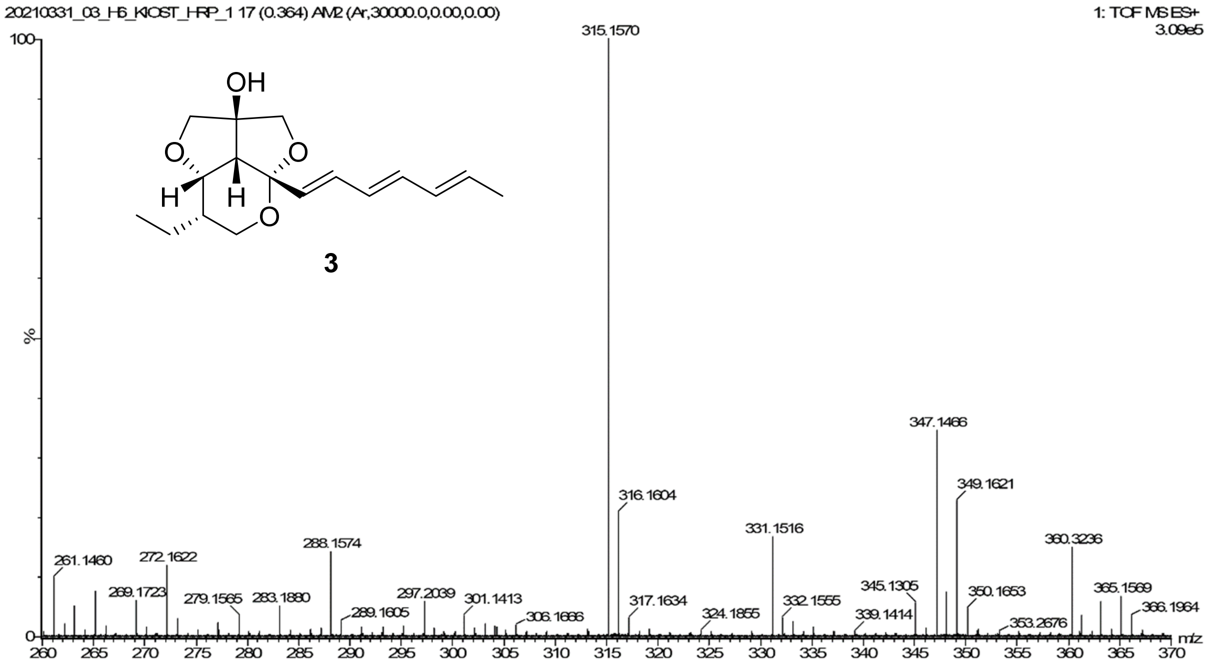

Figure S17. HRESIMS data of **3**.

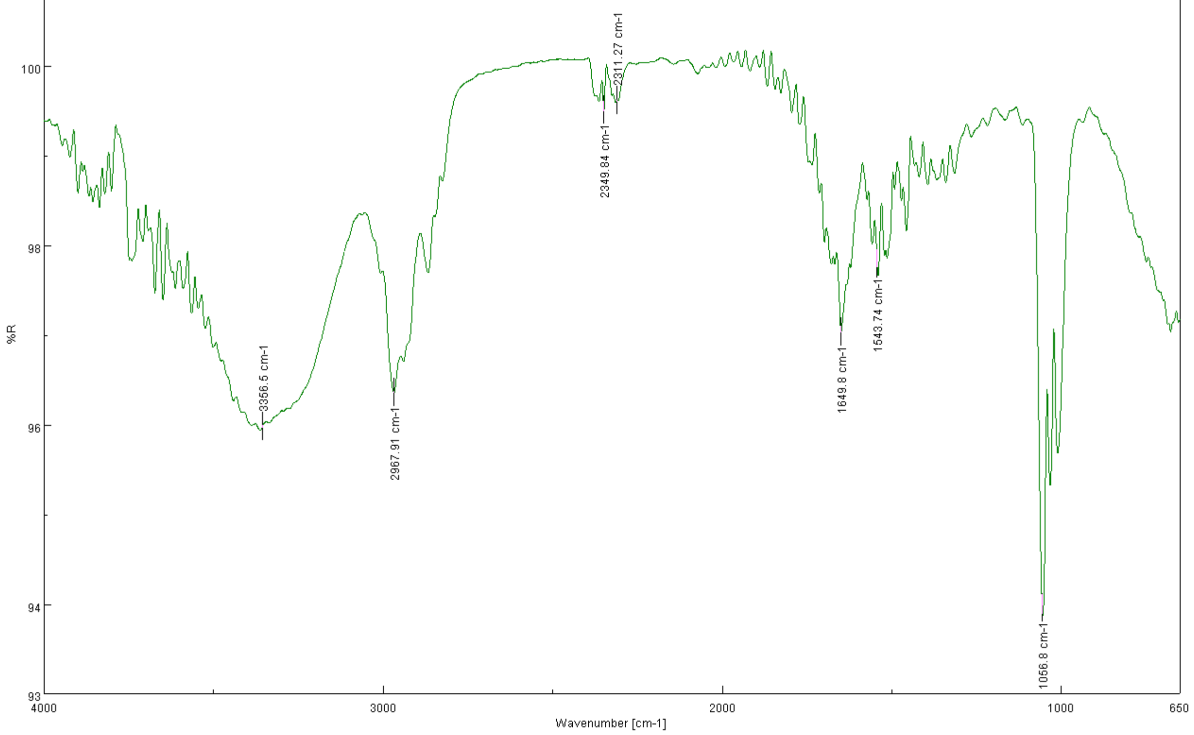

Figure S18. IR spectrum of **3**.

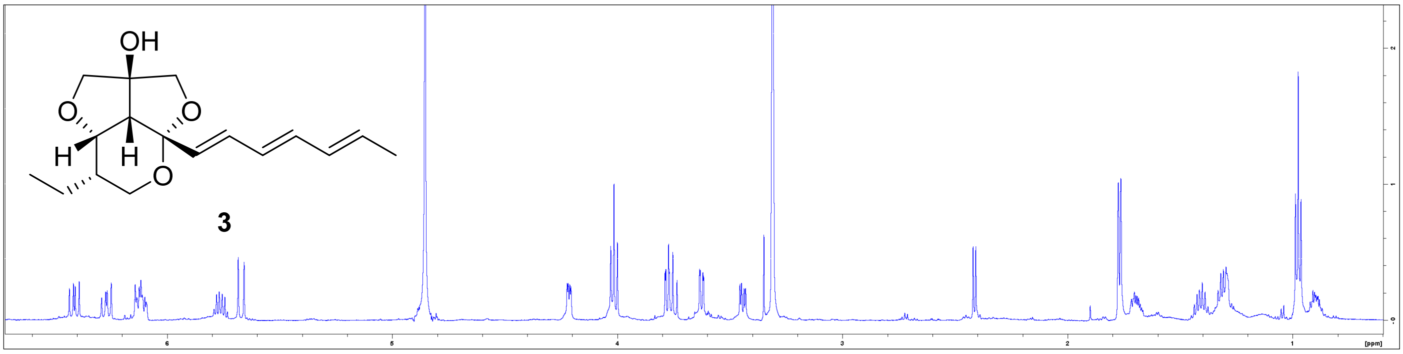

Figure S19. ^{1}H NMR spectrum of streptoglyceride G (**3**) in CD3OD (600MHz).

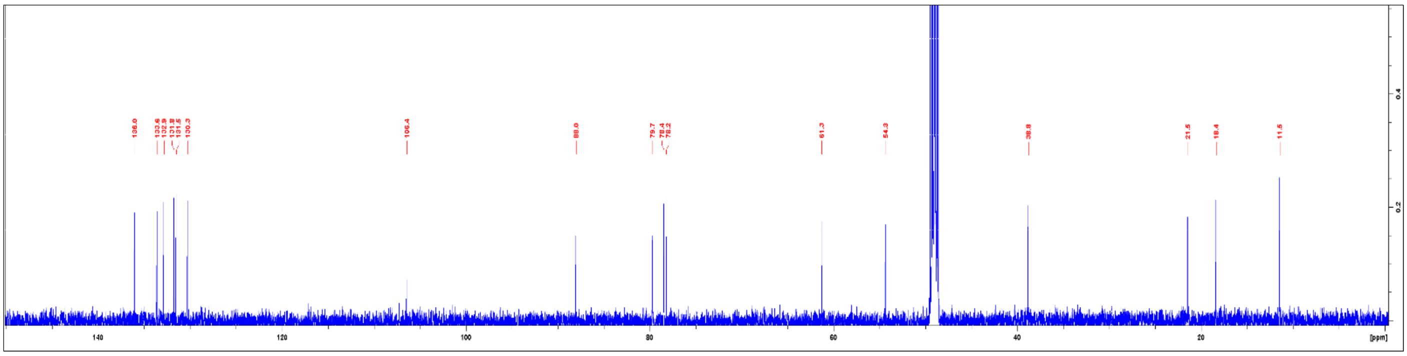

Figure S20. ^{13}C NMR spectrum of streptoglyceride G (**3**) in CD3OD (150MHz).

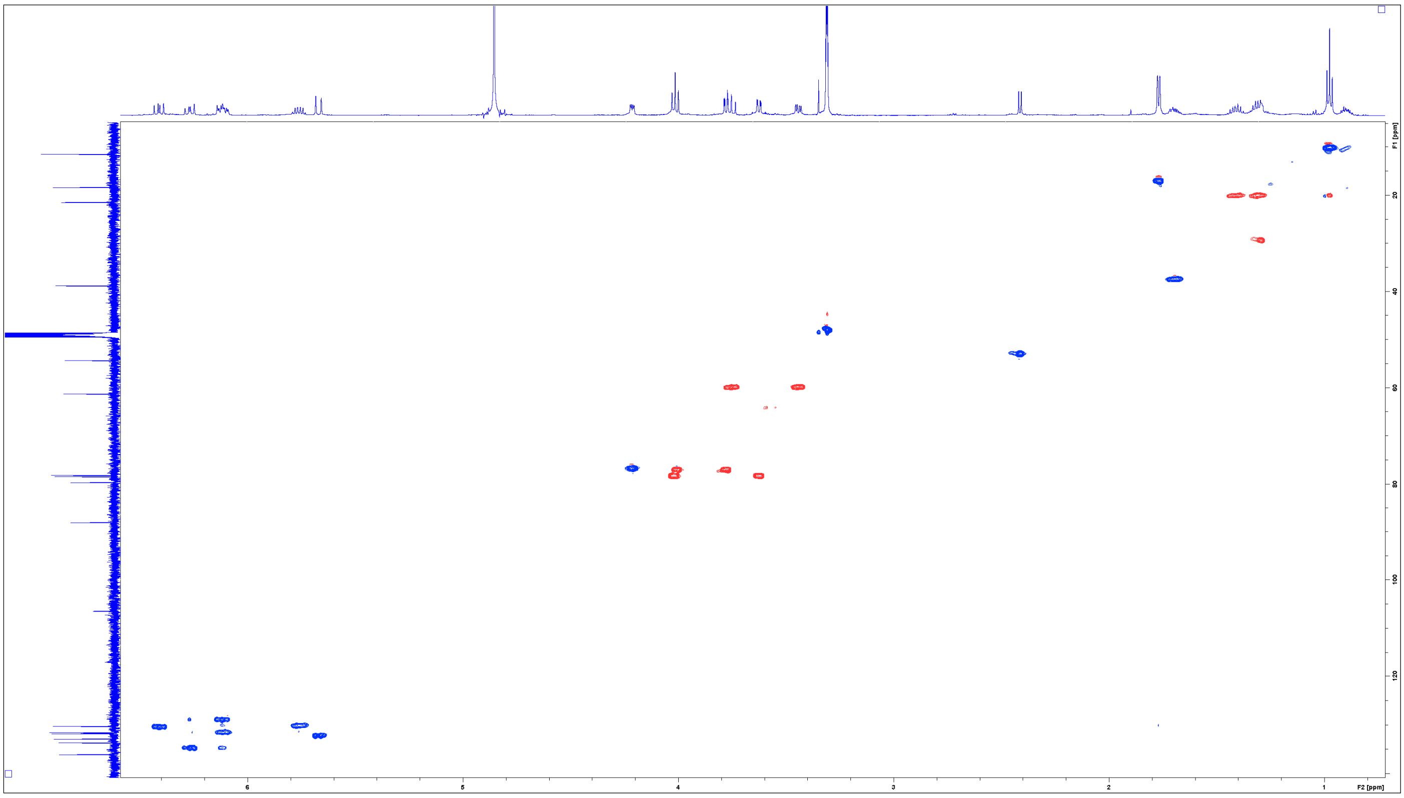

Figure S21. HSQC spectrum of streptoglyceride G (**3**) in CD_3OD (^{1}H: 600MHz, ^{13}C: 150MHz).

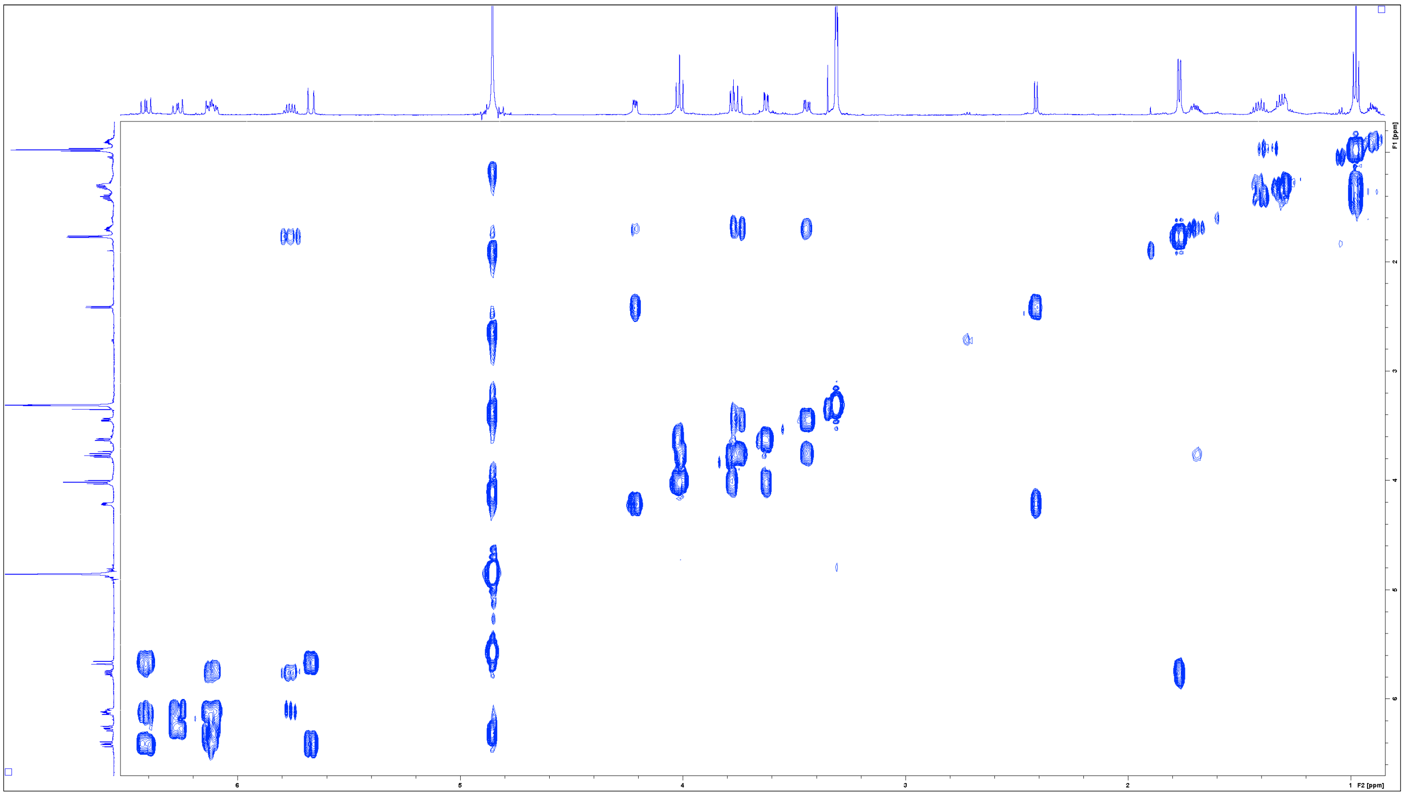

Figure S22. COSY spectrum of streptoglyceride G (**3**) in CD_3OD (^{1}H: 600MHz).

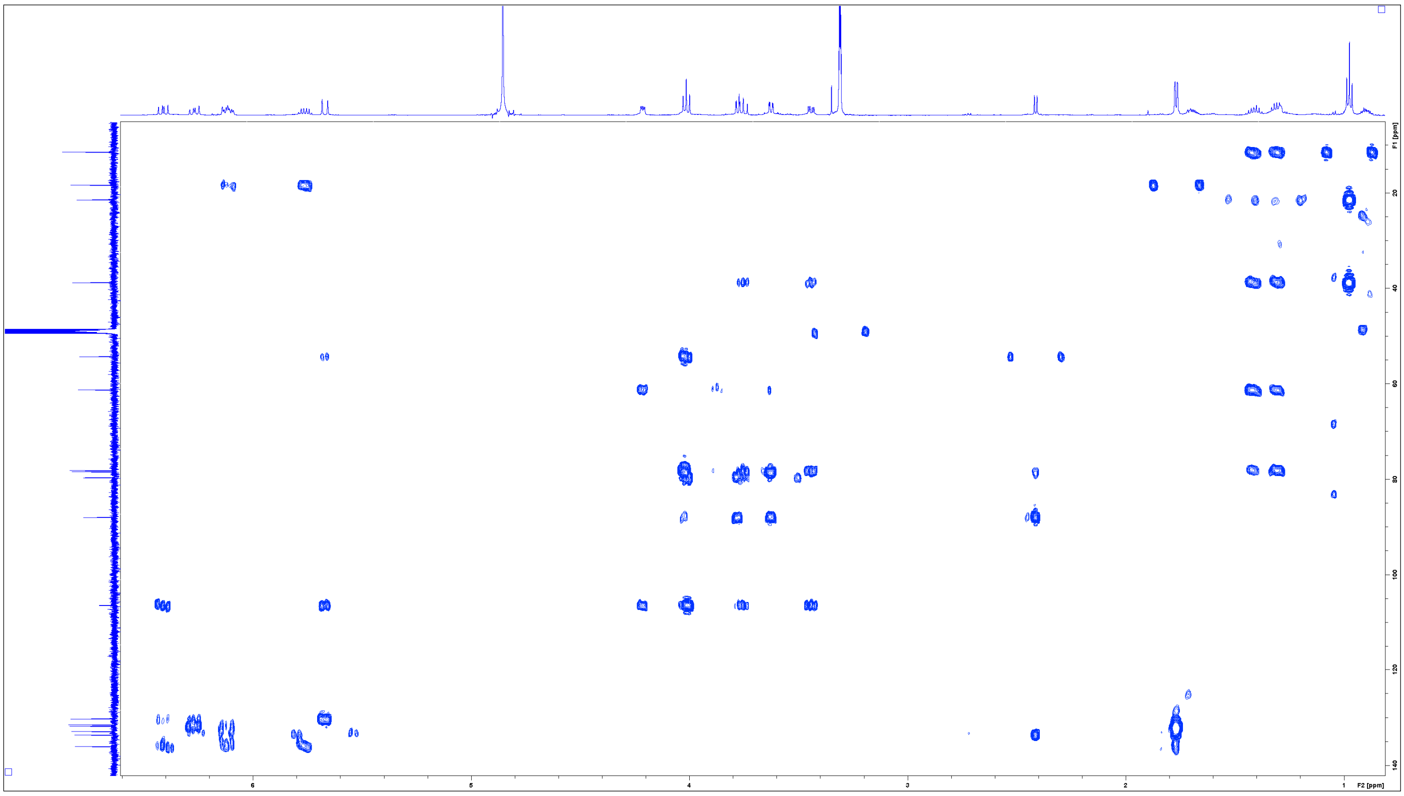

Figure S23. HMBC spectrum of streptoglyceride G (**3**) in CD_3OD (^{1}H: 600MHz, ^{13}C: 150MHz).

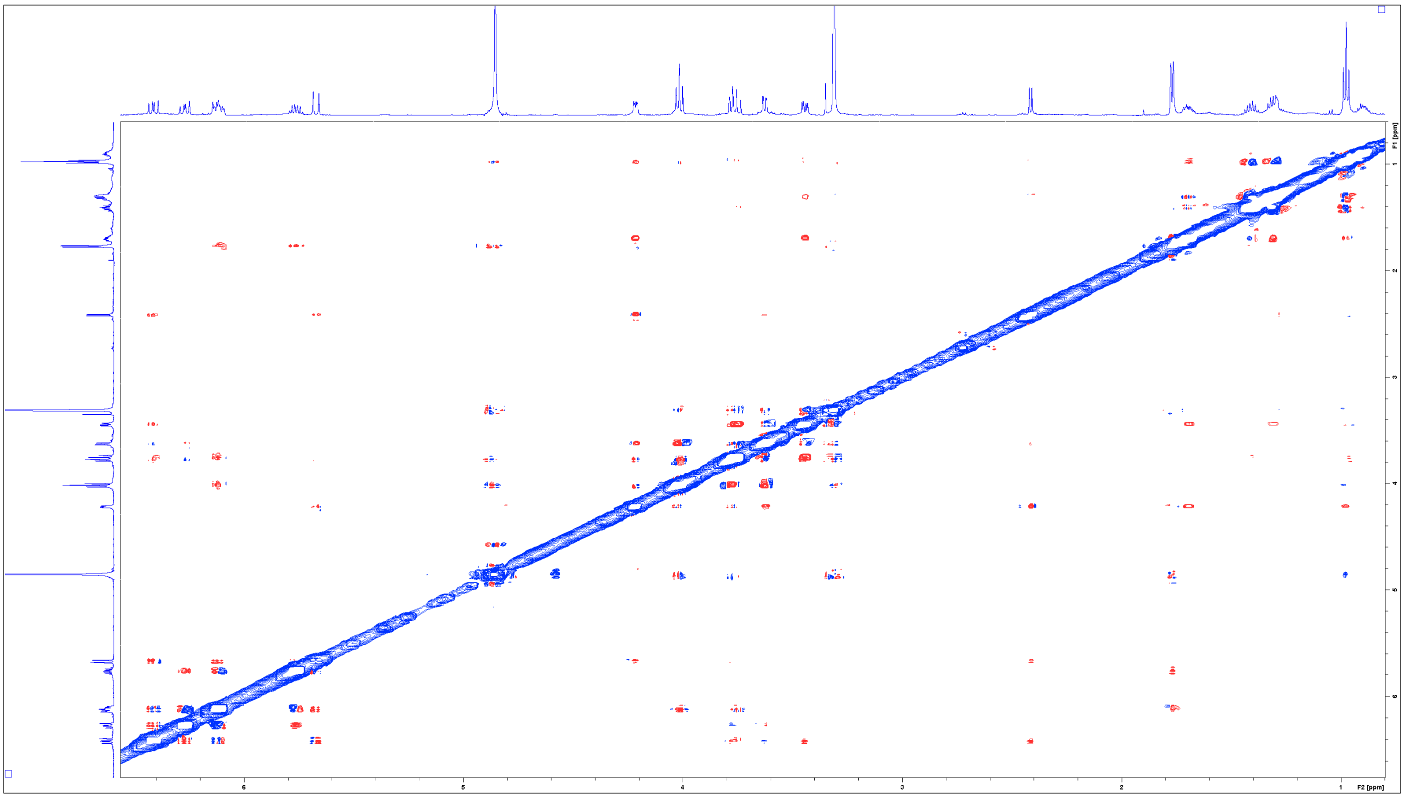

Figure S24. NOESY spectrum of streptoglyceride G (**3**) in CD_3OD (^{1}H: 600MHz).

Elemental Composition Report

Single Mass Analysis
Tolerance = 5.0 PPM / DBE: min = -1.5, max = 100.0
Element prediction: Off
Number of isotope peaks used for i-FIT = 3

Monoisotopic Mass, Even Electron Ions
39 formula(e) evaluated with 1 results within limits (all results (up to 1000) for each mass)
Elements Used:
C: 0-20 H: 0-30 O: 0-5 Na: 0-1

Minimum:				-1.5				
Maximum:		100.0	5.0	100.0				
Mass	Calc. Mass	mDa	PPM	DBE	i-FIT	Norm	Conf(%)	Formula
315.1573	315.1572	0.1	0.3	5.5	1268.2	n/a	n/a	C17 H24 O4 Na

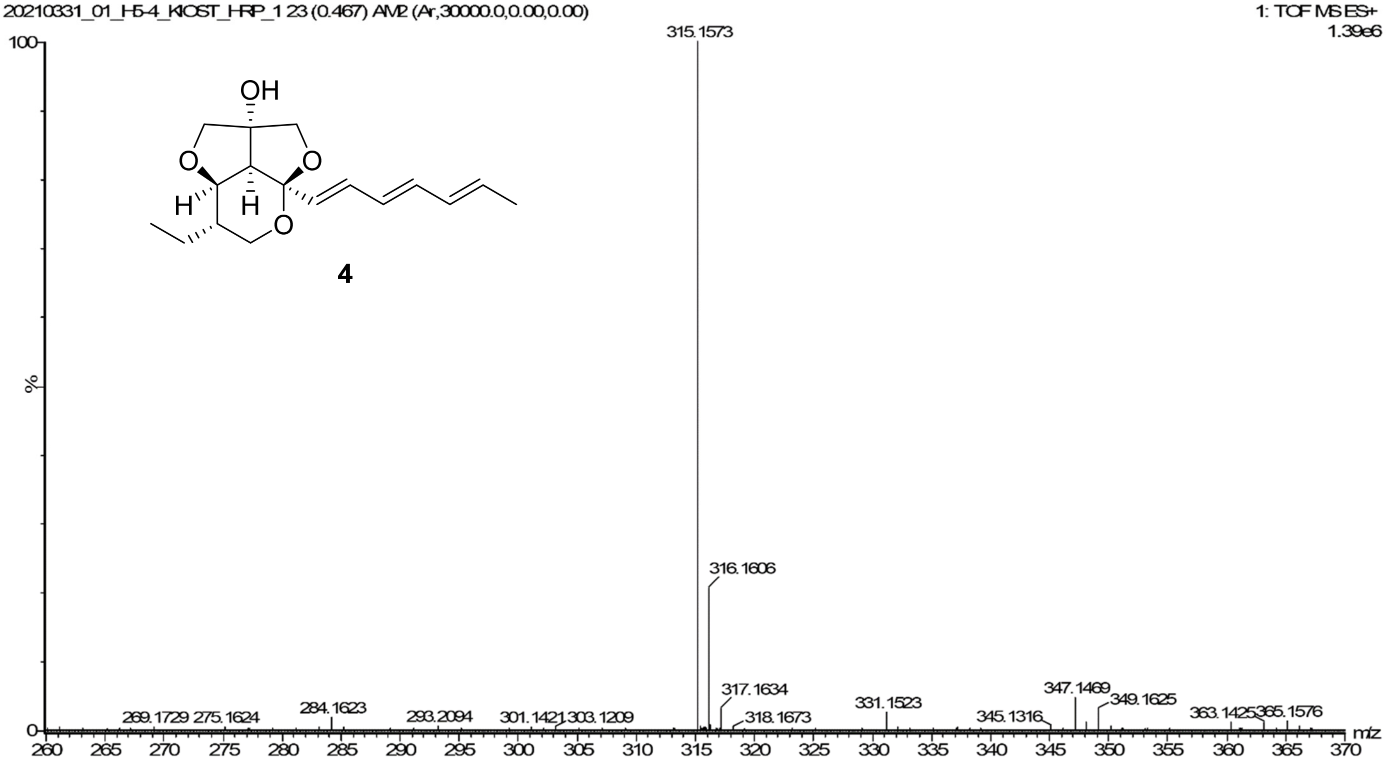

Figure S25. HRESIMS data of **4**.

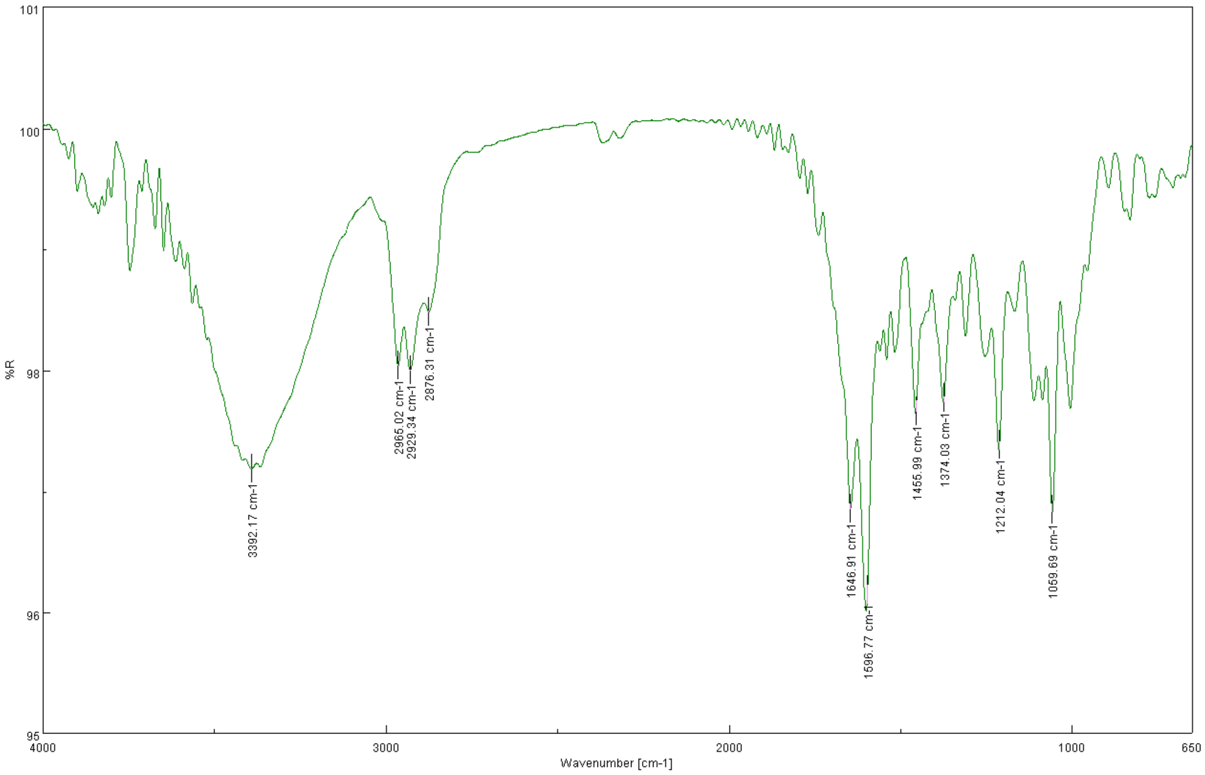

Figure S26. IR spectrum of **4**.

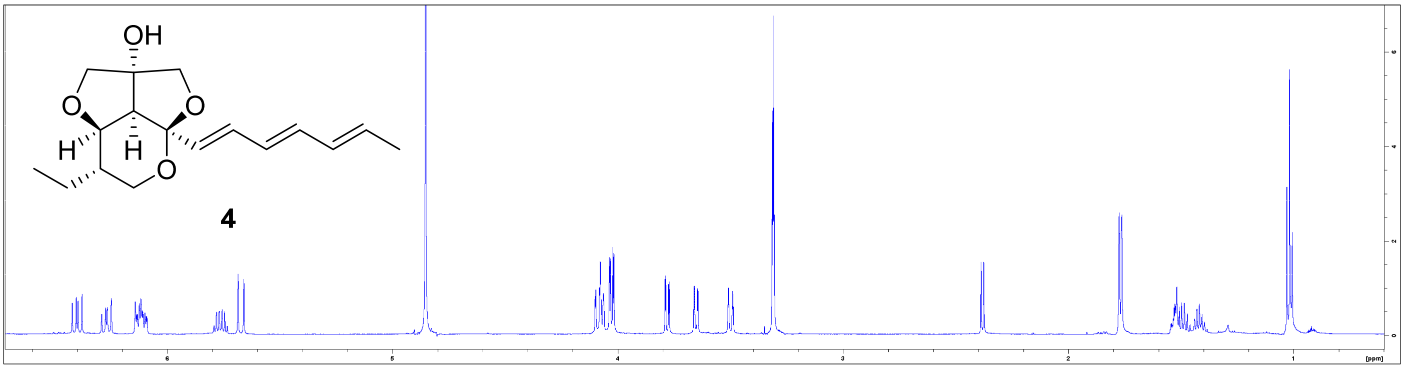

Figure S27. ^{1}H NMR spectrum of streptoglyceride H (**4**) in CD_3OD (600MHz).

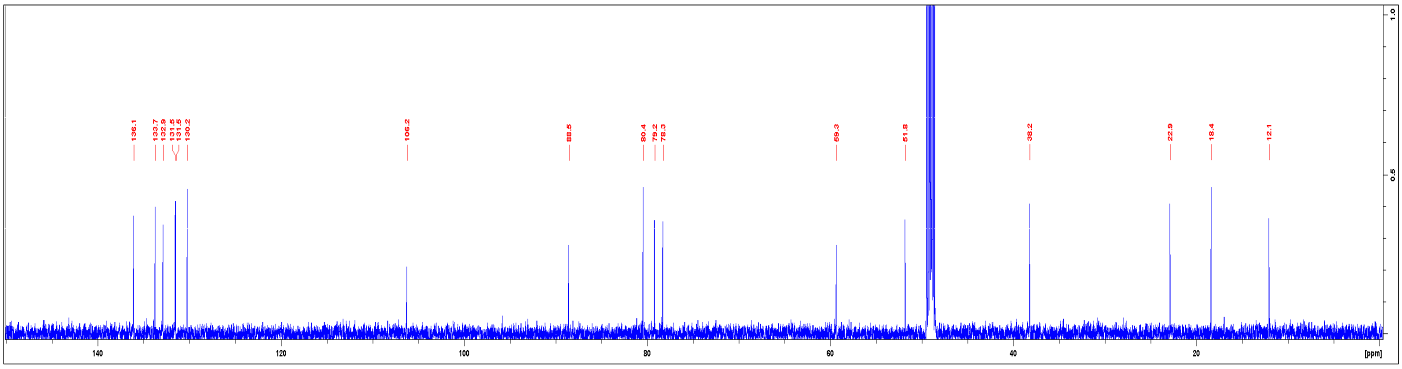

Figure S28. ^{13}C NMR spectrum of streptoglyceride H (**4**) in CD_3OD (150MHz).

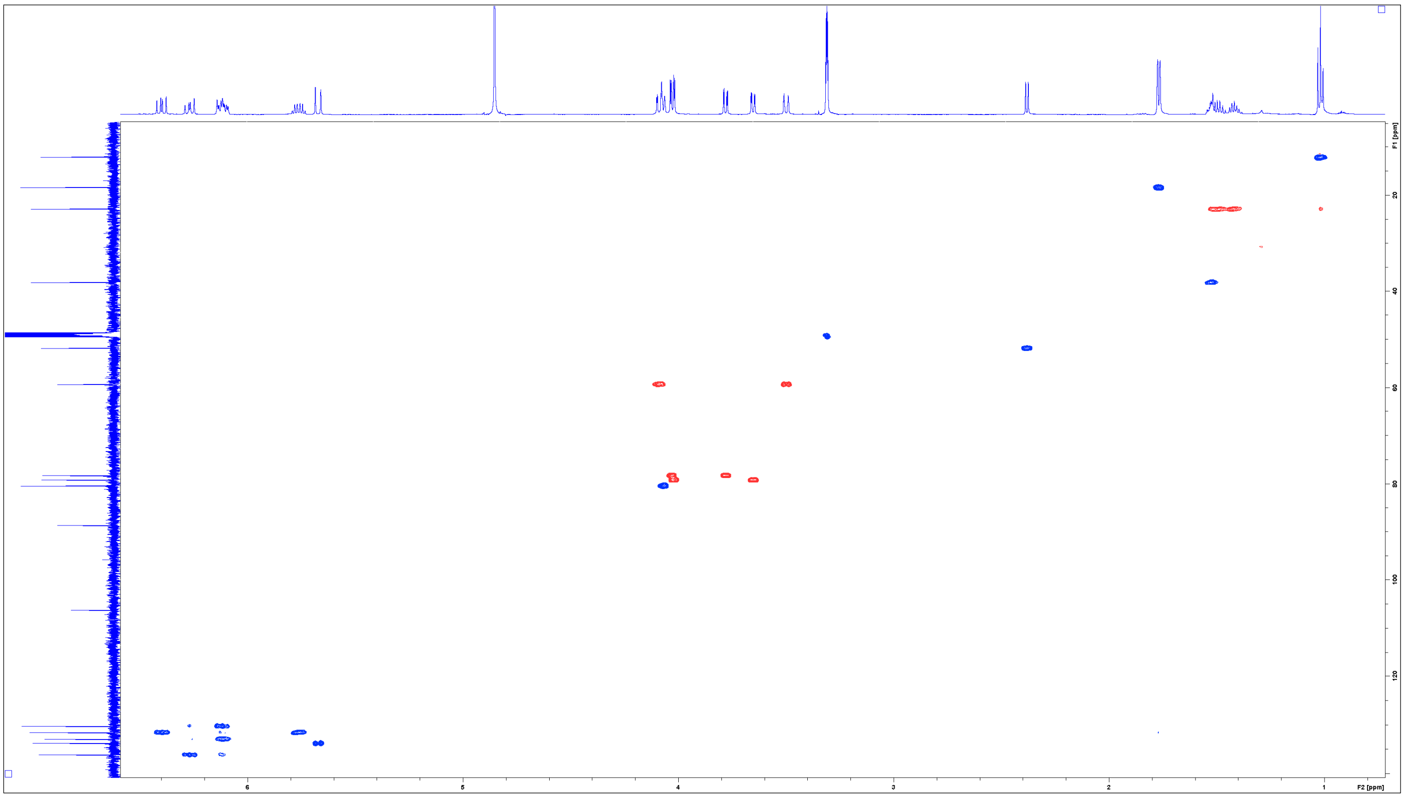

Figure S29. HSQC spectrum of streptoglyceride H (**4**) in CD_3OD (^{1}H: 600MHz, ^{13}C: 150MHz).

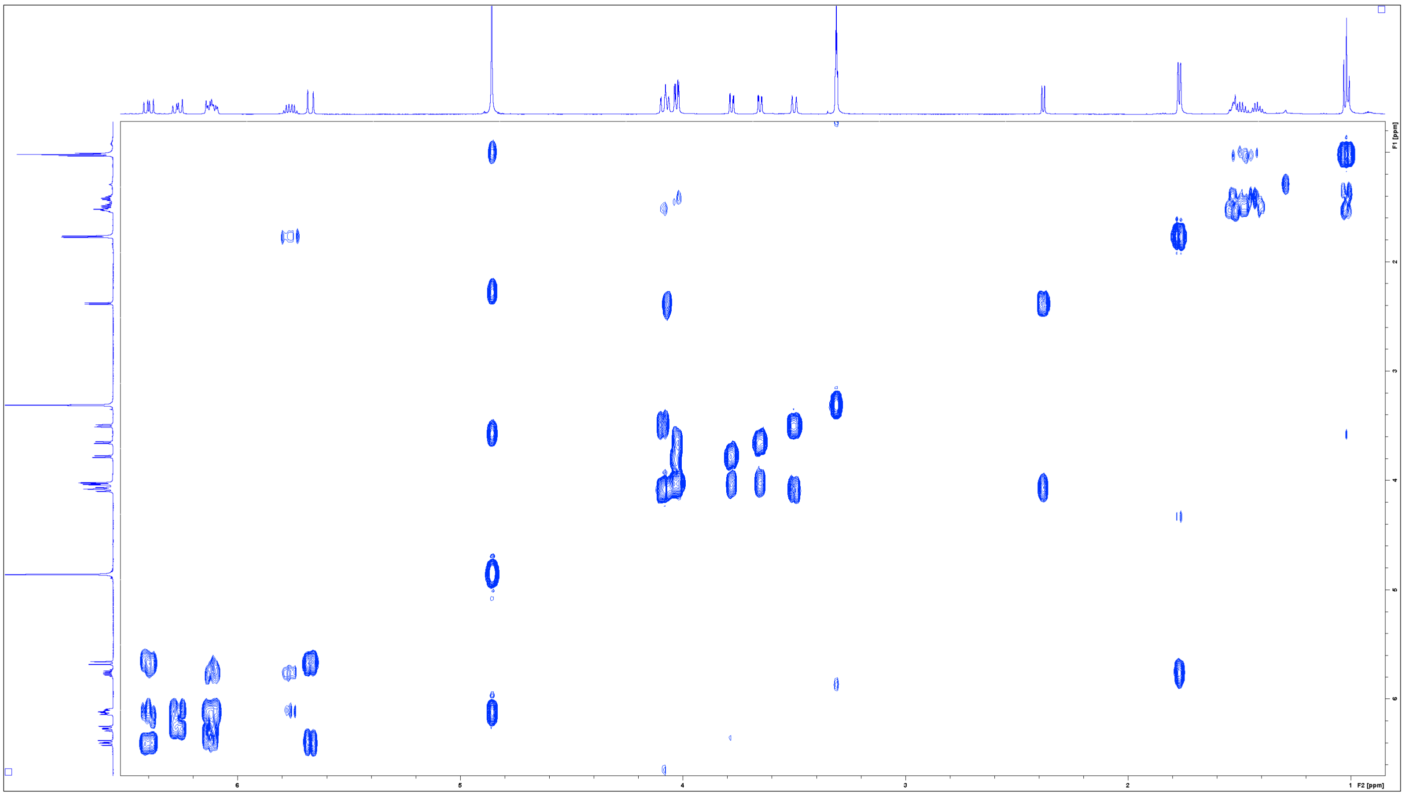

Figure S30. COSY spectrum of streptoglyceride H (**4**) in CD_3OD (^{1}H: 600MHz).

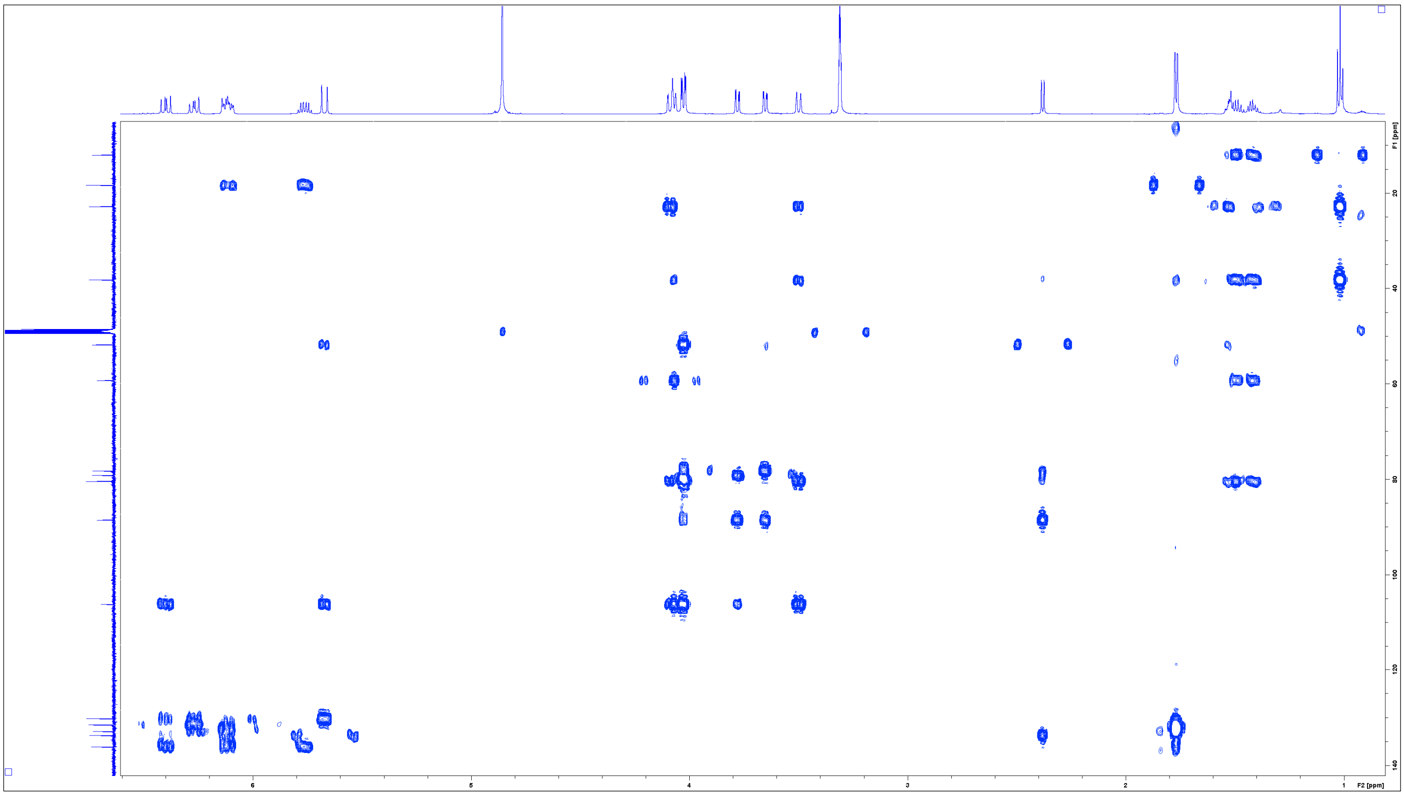

Figure S31. HMBC spectrum of streptoglyceride H (**4**) in CD3OD (^{1}H: 600MHz, ^{13}C: 150MHz).

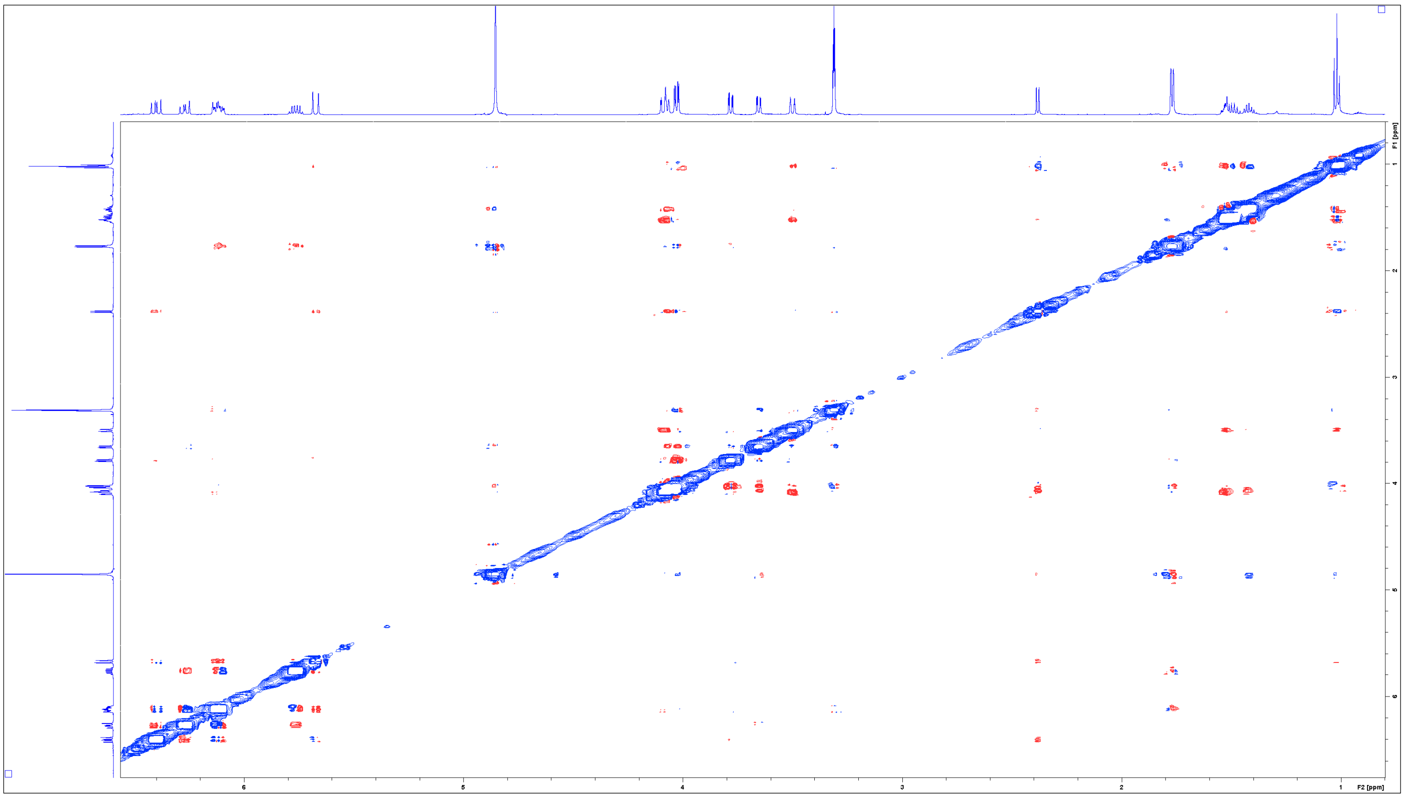

Figure S32. NOESY spectrum of streptoglyceride H (**4**) in CD3OD (^{1}H: 600MHz).

Table S1. Experimental chemical shifts and calculated chemical shifts of **1** and **1′** for DP4+ calculation

Atom	Experimental δ of **1**	Calculated δ of conformer **1**	Calculated δ of conformer **1′**
C	62.3	62.5	73.1
C	32.1	36.9	35.8
C	80.0	82.4	74.8
C	54.3	56.2	59.4
C	106.1	109.0	110.4
C	133.6	139.4	132.1
C	131.8	135.8	139.6
C	130.3	132.2	133.8
C	136.0	140.0	143.2
C	132.9	136.3	136.9
C	131.5	143.9	143.3
C	18.4	23.3	20.4
C	79.7	84.5	74.5
C	87.4	88.9	93.5
C	78.4	77.6	77.8
C	13.0	15.9	14.3
H	3.36	3.63	4.20
H	3.74	4.11	4.47
H	1.92	2.12	2.53
H	4.11	4.28	5.33
H	2.41	2.68	3.71
H	5.66	5.83	6.42
H	6.40	6.82	7.18
H	6.11	6.44	7.16
H	6.26	6.66	7.35
H	6.10	6.64	7.21
H	5.75	6.31	6.98
H	1.76	2.03	2.48
H	1.76	2.23	2.57
H	1.76	2.23	2.57
H	3.62	3.99	4.50
H	4.02	4.43	4.53
H	3.76	3.92	4.51
H	4.00	4.18	4.57
H	0.94	1.17	1.30
H	0.94	1.23	1.90
H	0.94	1.41	2.27

F12 =Main!G11

	A	B	C	D	E	F	G	H
1	Functional		Solvent?		Basis Set		Type of Data	
2	B3LYP		PCM		6-311+G(d,p)		Unscaled Shifts	
3								
4			Isomer 1	Isomer 2	Isomer 3	Isomer 4	Isomer 5	Isomer 6
5	sDP4+ (H data)		100.00%	0.00%	-	-	-	-
6	sDP4+ (C data)		100.00%	0.00%	-	-	-	-
7	sDP4+ (all data)		100.00%	0.00%	-	-	-	-
8	uDP4+ (H data)		100.00%	0.00%	-	-	-	-
9	uDP4+ (C data)		100.00%	0.00%	-	-	-	-
10	uDP4+ (all data)		100.00%	0.00%	-	-	-	-
11	DP4+ (H data)		100.00%	0.00%	-	-	-	-
12	DP4+ (C data)		100.00%	0.00%	-	-	-	-
13	DP4+ (all data)		100.00%	0.00%	-	-	-	-

Figure S33. Result sheet of DP4+ probability calculations of isomers of **1**.

Isomer 1: 2*S**, 3*S**, 4*R**, 5S*, 14S* (**1**), Isomer 2: 2*S**, 3*S**, 4*R**, 5S*, 14R* (**1'**).

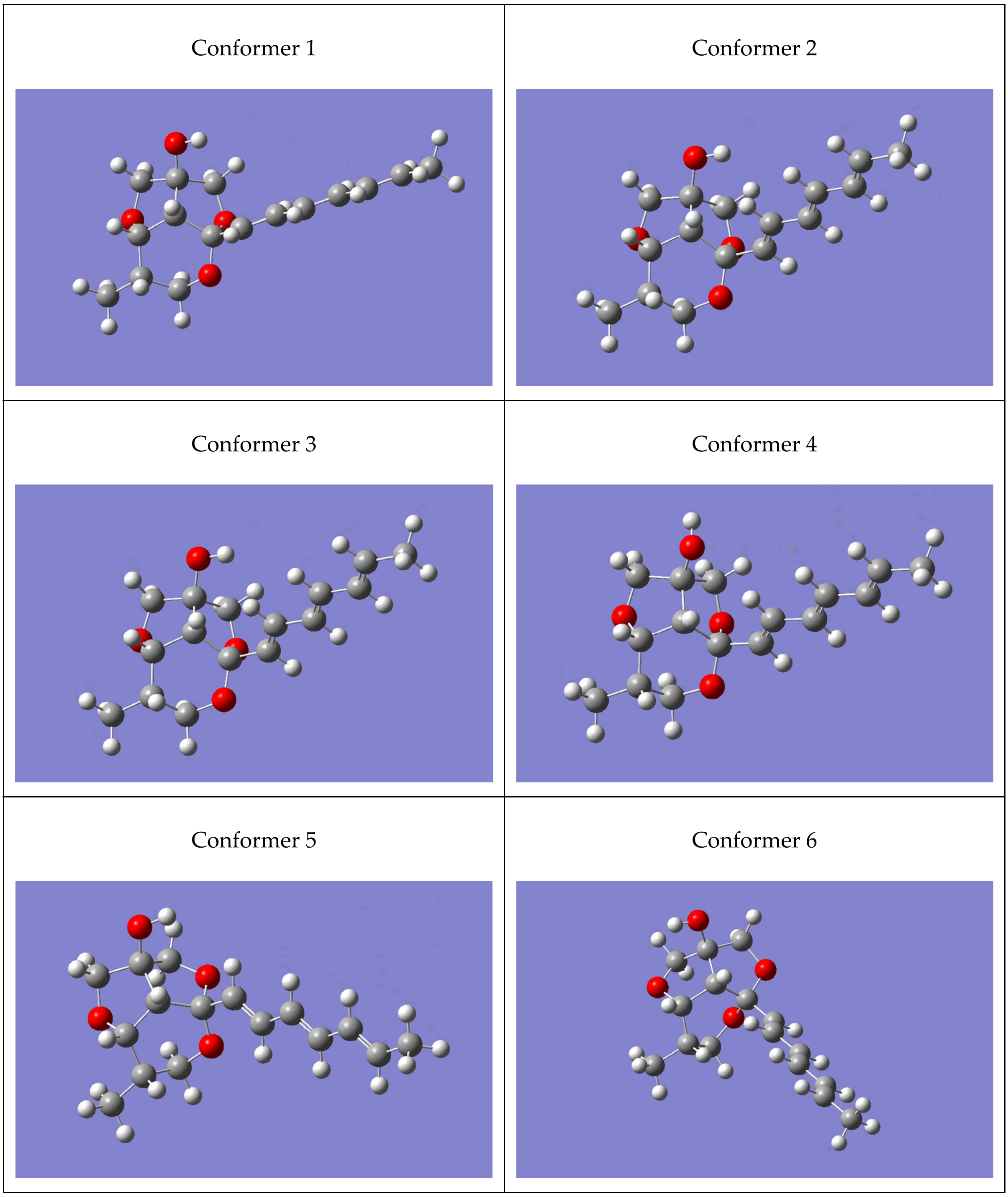

Figure S34. Energy-minimized models of conformers of **1A**.

Table S2. Hartrees energy, and Boltzmann's distribution of conformers of **1A**

No	Energy	Boltzmann Pop
**1A** Conformer 1	-923.893093222	0.669649
**1A** Conformer 2	-923.891472018	0.120267
**1A** Conformer 3	-923.891470942	0.12013
**1A** Conformer 4	-923.890938163	0.0683266
**1A** Conformer 5	-923.889841732	0.0213928
**1A** Conformer 6	-923.885581679	0.000234845

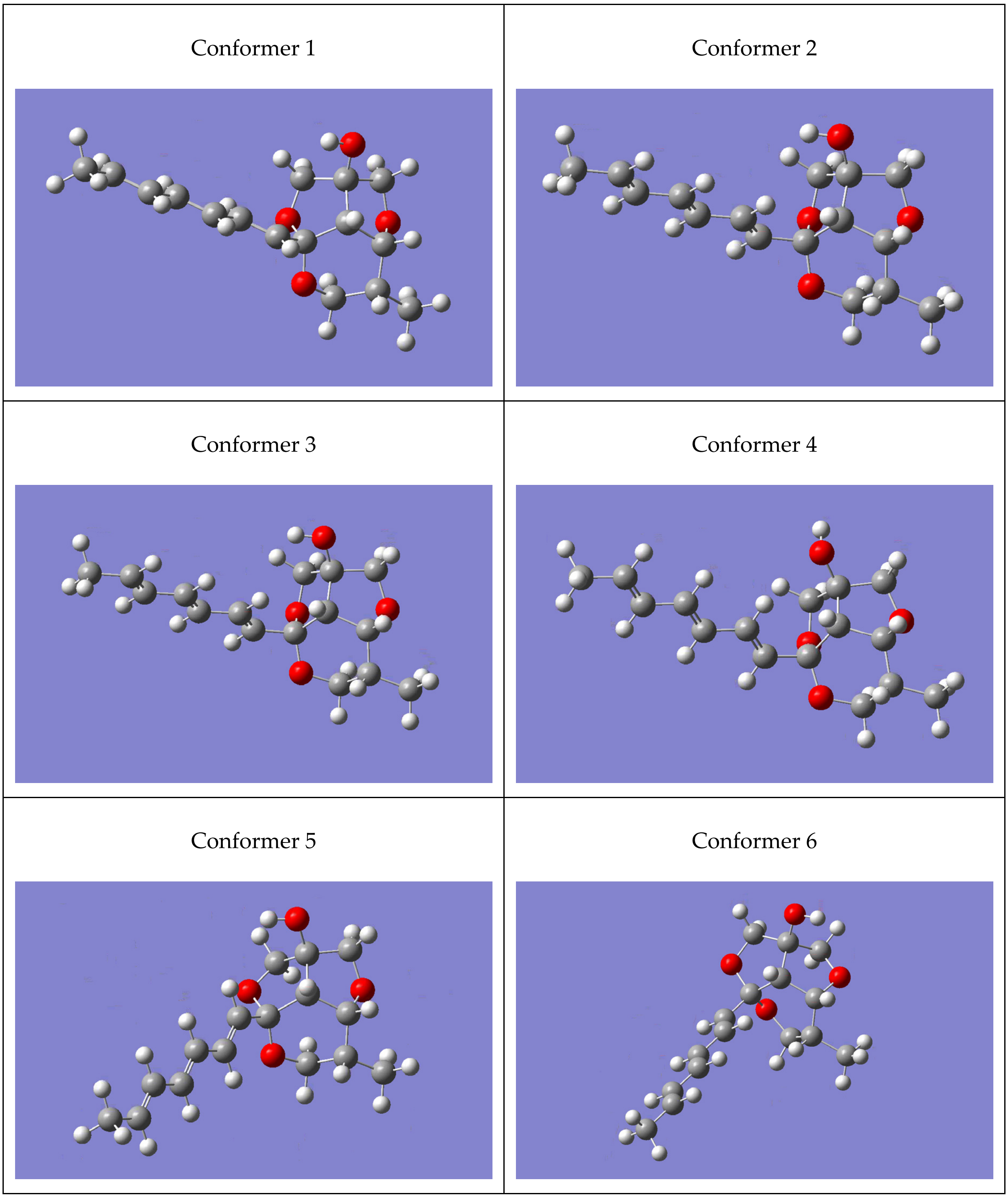

Figure S35. Energy-minimized models of conformers of **1B**.

Table S3. Hartrees energy, and Boltzmann's distribution of conformers of **1B**

No	Energy	Boltzmann Pop
**1B** Conformer 1	-923.893093222	0.669653
**1B** Conformer 2	-923.891472018	0.120268
**1B** Conformer 3	-923.891470896	0.120125
**1B** Conformer 4	-923.890938163	0.068327
**1B** Conformer 5	-923.889841732	0.0213929
**1B** Conformer 6	-923.885581679	0.000234846

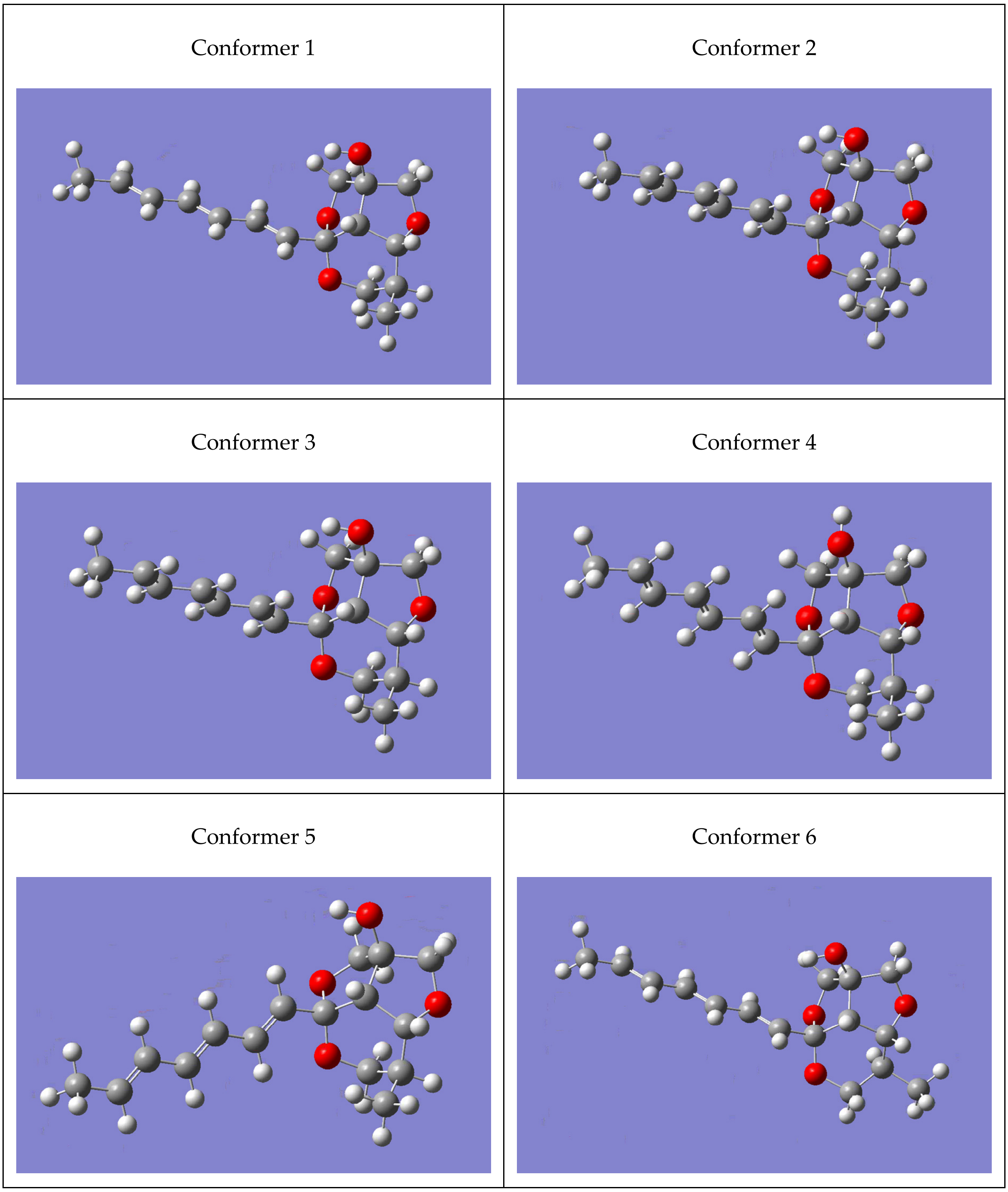

Figure S36. Energy-minimized models of conformers of **2A**.

Table S4. Hartrees energy, and Boltzmann's distribution of conformers of **2A**

No	Energy	Boltzmann Pop
**2A** Conformer 1	-923.892644801	0.664511
**2A** Conformer 2	-923.89100913	0.117529
**2A** Conformer 3	-923.891007591	0.117338
**2A** Conformer 4	-923.890471968	0.0665379
**2A** Conformer 5	-923.889595136	0.0262878
**2A** Conformer 6	-923.888447525	0.00779634

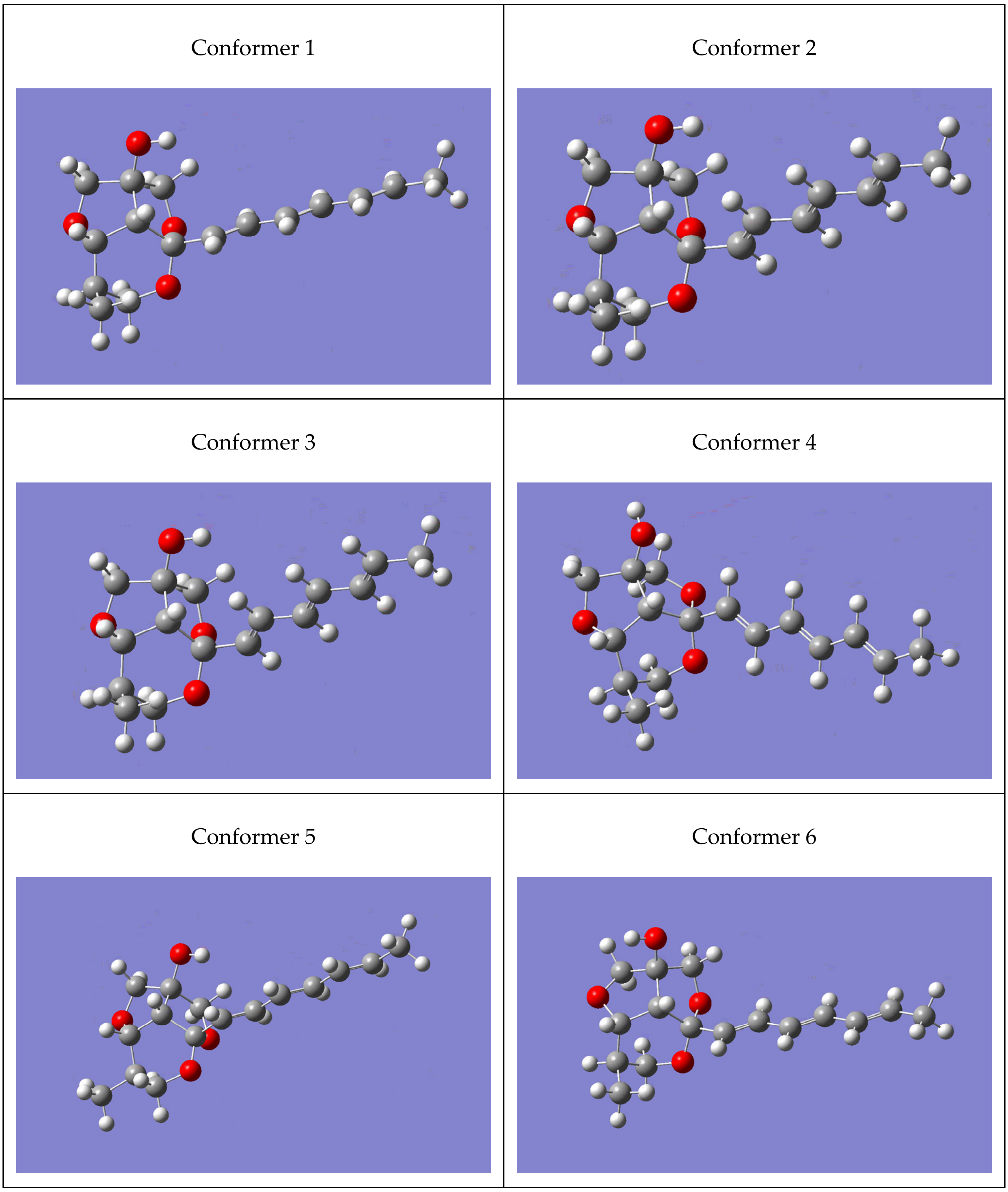

Figure S37. Energy-minimized models of conformers of **2B**.

Table S5. Hartrees energy, and Boltzmann's distribution of conformers of **2B**

No	Energy	Boltzmann Pop
**2B** Conformer 1	-923.892644671	0.704034
**2B** Conformer 2	-923.891009168	0.124542
**2B** Conformer 3	-923.891007544	0.124328
**2B** Conformer 4	-923.889824467	0.0355133
**2B** Conformer 5	-923.888447525	0.00826118
**2B** Conformer 6	-923.887587321	0.00332182

Conformer 1	Conformer 2
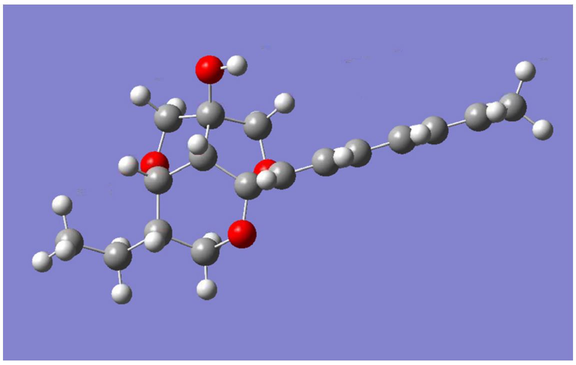	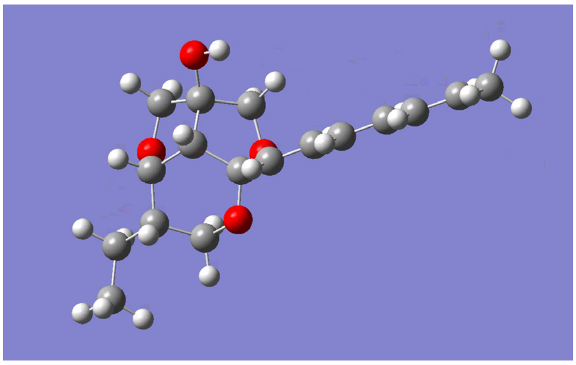
Conformer 3	Conformer 4
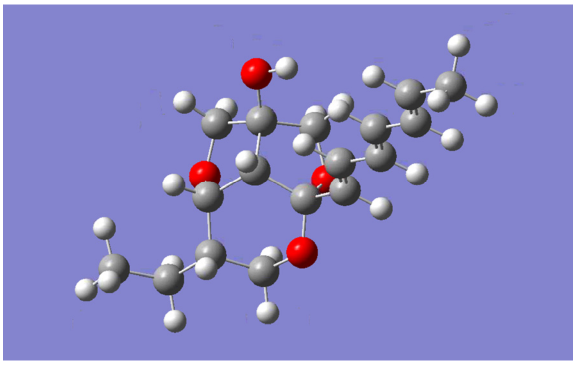	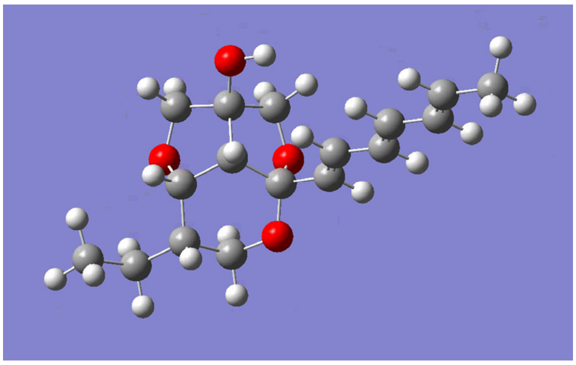
Conformer 5	Conformer 6
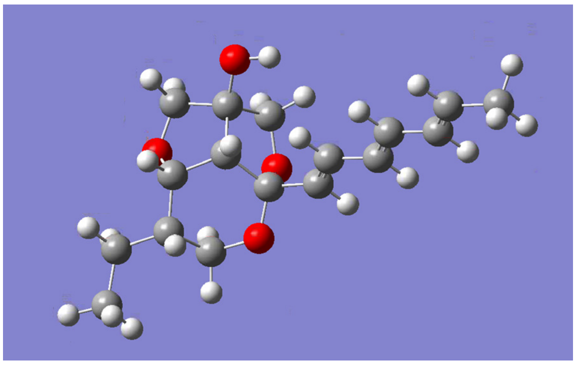	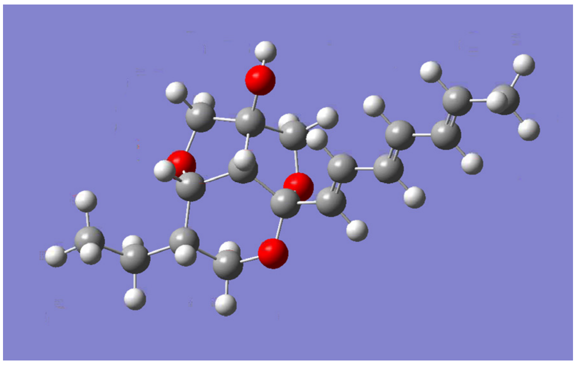

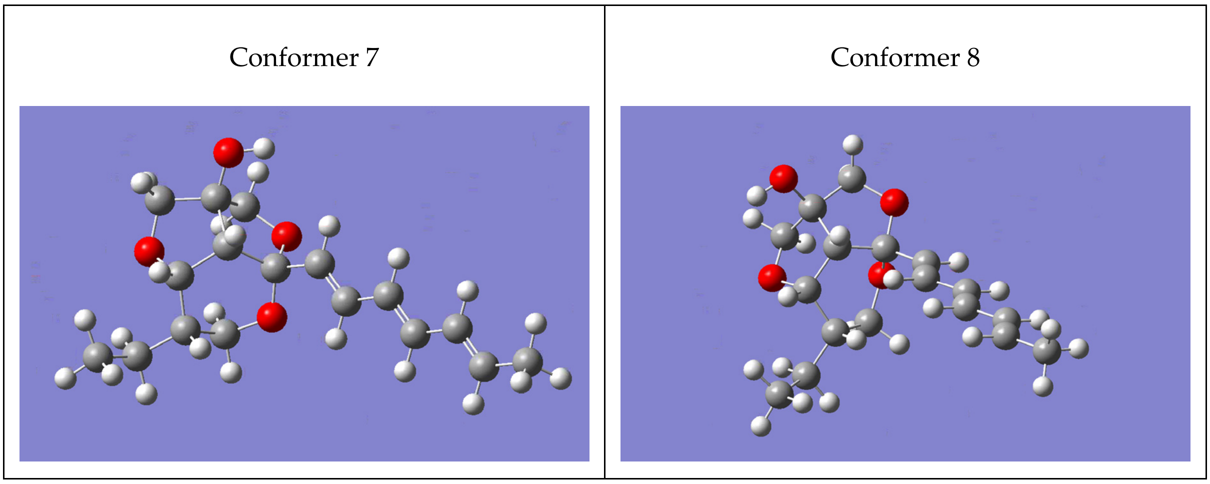

Figure S38. Energy-minimized models of conformers of **3A**.

Table S6. Hartrees energy, and Boltzmann's distribution of conformers of **3A**

No	Energy	Boltzmann Pop
**3A** Conformer 1	-963.216103841	0.411829
**3A** Conformer 2	-963.215925708	0.341021
**3A** Conformer 3	-963.214411687	0.0686089
**3A** Conformer 4	-963.214411648	0.0686061
**3A** Conformer 5	-963.214188559	0.0541688
**3A** Conformer 6	-963.213953978	0.0422522
**3A** Conformer 7	-963.21286717	0.0133645
**3A** Conformer 8	-963.208627218	0.000149869

Conformer 1	Conformer 2
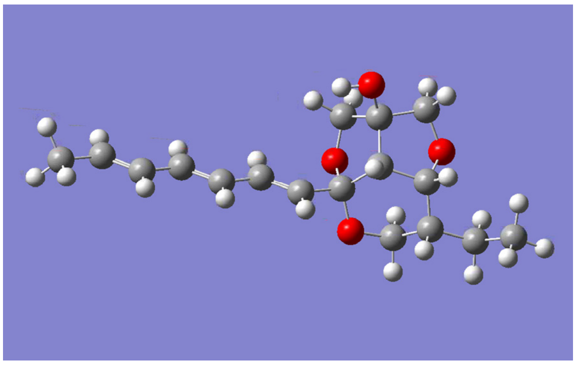	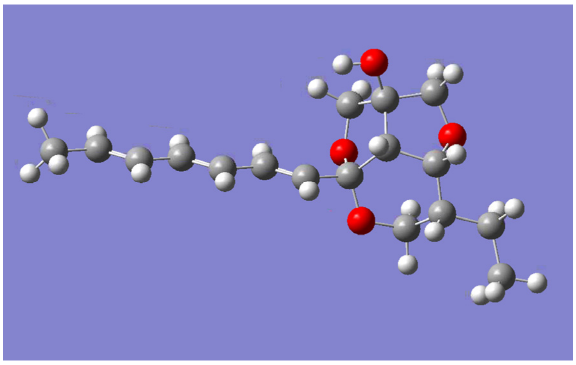
Conformer 3	Conformer 4
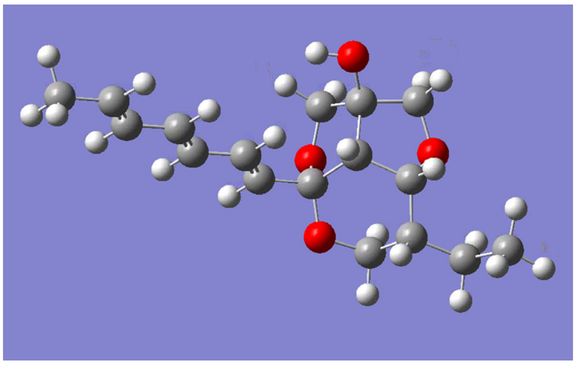	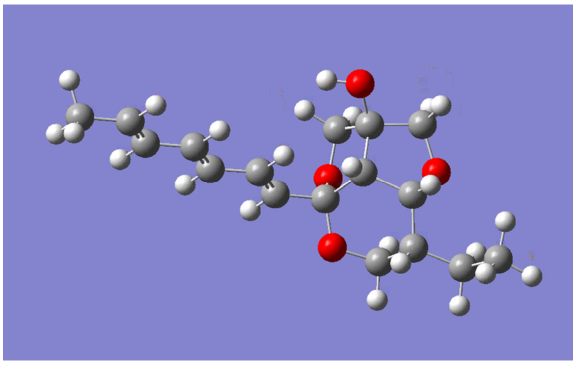
Conformer 5	Conformer 6
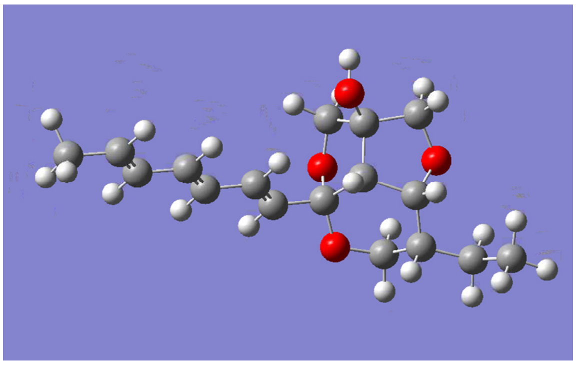	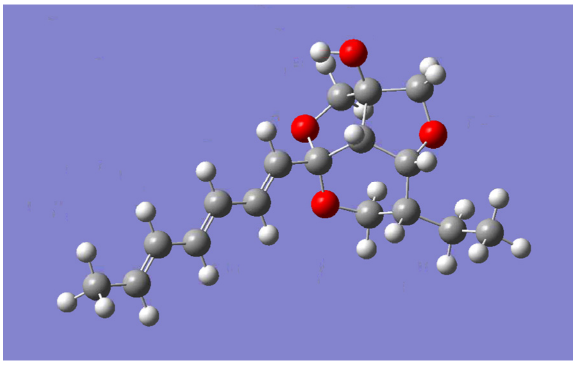

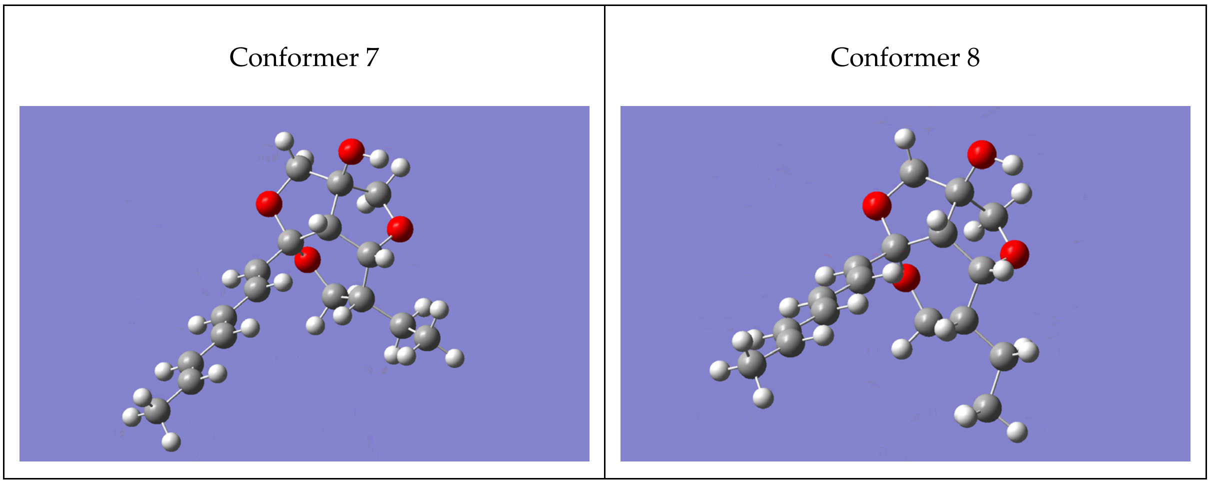

Figure S39. Energy-minimized models of conformers of **3B**.

Table S7. Hartrees energy, and Boltzmann's distribution of conformers of **3B**

No	Energy	Boltzmann Pop
**3B** Conformer 1	-963.216103841	0.435359
**3B** Conformer 2	-963.215925708	0.360506
**3B** Conformer 3	-963.214411785	0.0725365
**3B** Conformer 4	-963.214411649	0.0725261
**3B** Conformer 5	-963.213953978	0.0446663
**3B** Conformer 6	-963.212867171	0.0141281
**3B** Conformer 7	-963.208627217	0.000158432
**3B** Conformer 8	-963.208357975	0.000119124

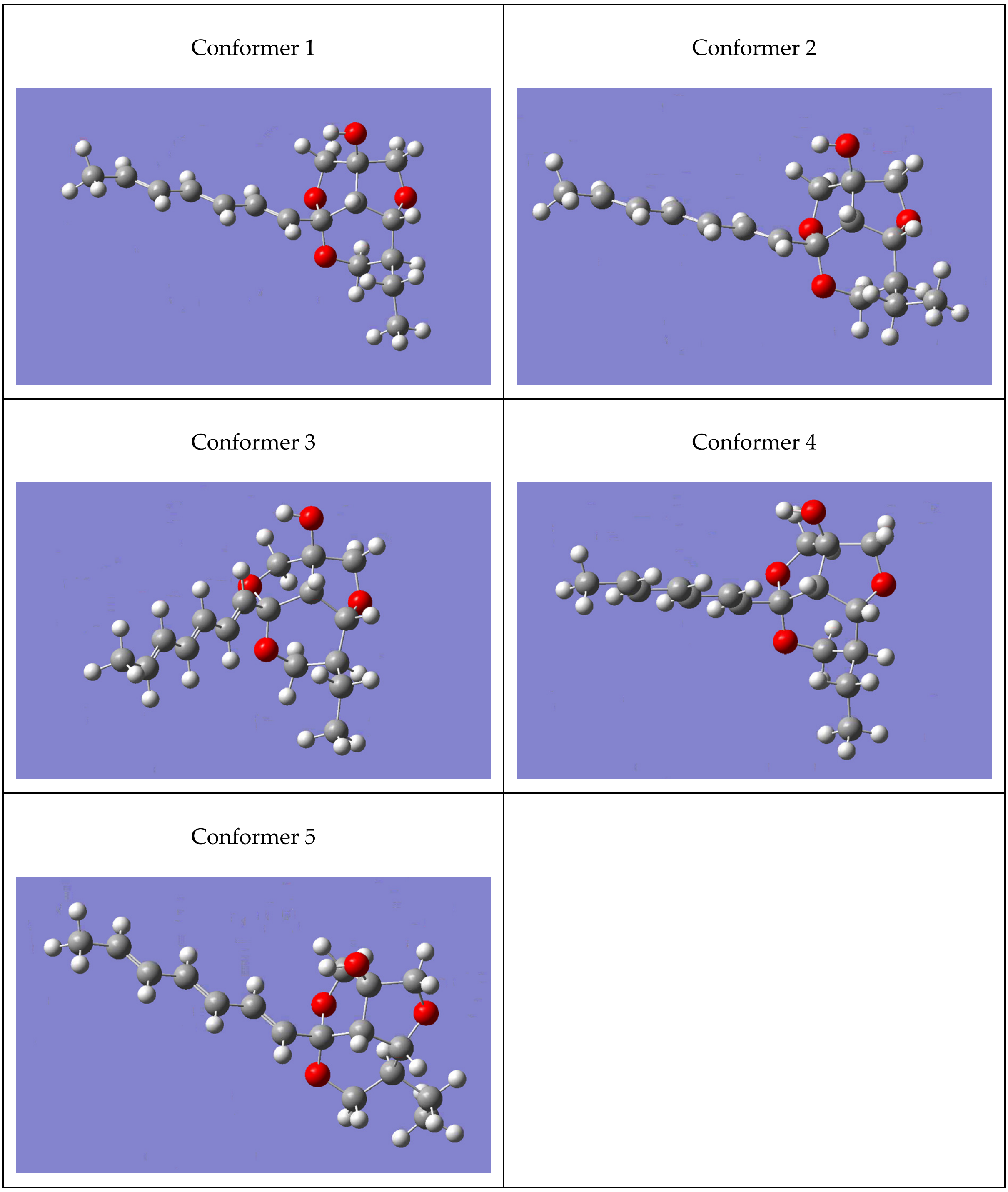

Figure S40. Energy-minimized models of conformers of **4A**.

Table S8. Hartrees energy, and Boltzmann's distribution of conformers of **4A**

No	Energy	Boltzmann Pop
**4A** Conformer 1	-963.215703217	0.606465
**4A** Conformer 2	-963.215200039	0.355926
**4A** Conformer 3	-963.21266132	0.0241898
**4A** Conformer 4	-963.21151303	0.00716895
**4A** Conformer 5	-963.211383453	0.00624963

Conformer 1

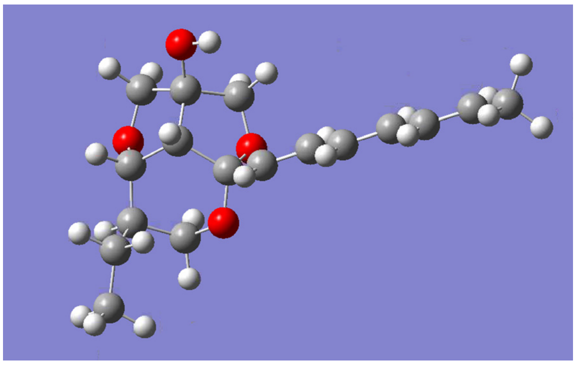

Conformer 2

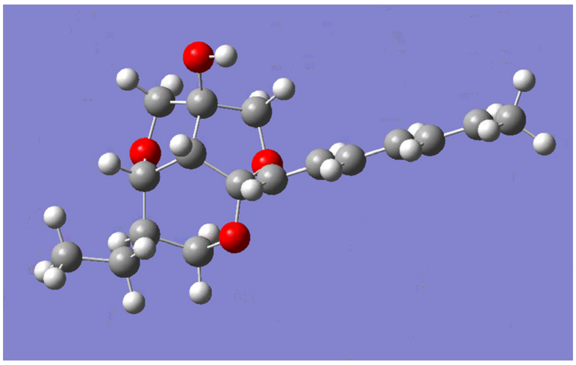

Conformer 3

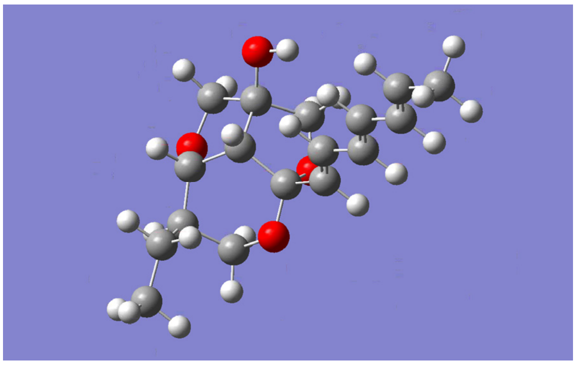

Conformer 4

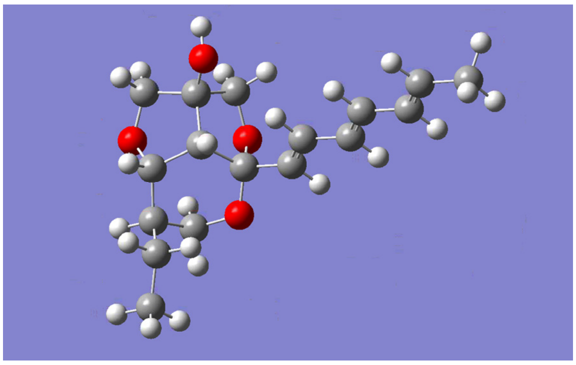

Conformer 5

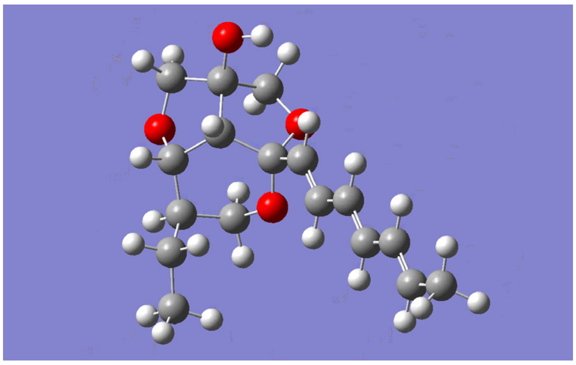

Figure S41. Energy-minimized models of conformers of **4B**.

Table S9. Hartrees energy, and Boltzmann's distribution of conformers of **4B**

No	Energy	Boltzmann Pop
**4B** Conformer 1	-963.215703328	0.530458
**4B** Conformer 2	-963.21520021	0.311338
**4B** Conformer 3	-963.214011579	0.0884099
**4B** Conformer 4	-963.21344736	0.0486383
**4B** Conformer 5	-963.21266132	0.0211556
